# Acid-Induced Type VI Secretion System Is Regulated by ExoR-ChvG/ChvI Signaling Cascade in *Agrobacterium tumefaciens*

**DOI:** 10.1371/journal.ppat.1002938

**Published:** 2012-09-27

**Authors:** Chih-Feng Wu, Jer-Sheng Lin, Gwo-Chyuan Shaw, Erh-Min Lai

**Affiliations:** 1 Institute of Plant and Microbial Biology, Academia Sinica, Taipei, Taiwan; 2 Institute of Biochemistry and Molecular Biology, National Yang-Ming University, Taipei, Taiwan; Massachusetts General Hospital, Harvard Medical School, United States of America

## Abstract

The type VI secretion system (T6SS) is a widespread, versatile protein secretion system in pathogenic *Proteobacteria*. Several T6SSs are tightly regulated by various regulatory systems at multiple levels. However, the signals and/or regulatory mechanisms of many T6SSs remain unexplored. Here, we report on an acid-induced regulatory mechanism activating T6SS in *Agrobacterium tumefaciens*, a plant pathogenic bacterium causing crown gall disease in a wide range of plants. We monitored the secretion of the T6SS hallmark protein hemolysin-coregulated protein (Hcp) from *A. tumefaciens* and found that acidity is a T6SS-inducible signal. Expression analysis of the T6SS gene cluster comprising the *imp* and *hcp* operons revealed that *imp* expression and Hcp secretion are barely detected in *A. tumefaciens* grown in neutral minimal medium but are highly induced with acidic medium. Loss- and gain-of-function analysis revealed that the *A. tumefaciens* T6SS is positively regulated by a *chvG/chvI* two-component system and negatively regulated by *exoR*. Further epistasis analysis revealed that *exoR* functions upstream of the *chvG* sensor kinase in regulating T6SS. ChvG protein levels are greatly increased in the *exoR* deletion mutant and the periplasmic form of overexpressed ExoR is rapidly degraded under acidic conditions. Importantly, ExoR represses ChvG by direct physical interaction, but disruption of the physical interaction allows ChvG to activate T6SS. The phospho-mimic but not wild-type ChvI response regulator can bind to the T6SS promoter region *in vitro* and activate T6SS with growth in neutral minimal medium. We present the first evidence of T6SS activation by an ExoR-ChvG/ChvI cascade and propose that acidity triggers ExoR degradation, thereby derepressing ChvG/ChvI to activate T6SS in *A. tumefaciens*.

## Author Summary

The bacterial type VI secretion system (T6SS) has diverse functions that contribute to the survival or fitness of many pathogenic bacteria in response to environmental cues. Numerous studies have shown that T6SS is highly regulated via multiple mechanisms, but the regulatory mechanisms of most T6SSs remain unknown. In this study, we discovered that T6SS is activated by acidity via an ExoR-ChvG/ChvI cascade in a plant pathogenic bacterium, *Agrobacterium tumefaciens*. Our data suggested that ExoR represses ChvG sensor kinase by physical interaction and the acid-induced degradation of periplasmic ExoR may derepress ChvG to activate T6SS by phosphorylation of the ChvI response regulator. The activation of T6SS by an acidic signal present in the wound site and intercellular space of plants implicates a role of T6SS during *Agrobacterium*–plant interactions. In view of the conservation of ExoR and ChvG/ChvI and wide distribution of T6SS in *α-Proteobacteria*, including many animal and plant pathogens and symbionts, the regulation of T6SS by the ExoR-ChvG/ChvI cascade may be a universal regulatory mechanism in these bacteria.

## Introduction

Pathogenic bacteria have evolved several specialized secretion systems to transport protein or DNA across membranes to extracellular milieu or even to the host cells in response to specific environmental cues. Among the 6 types of secretion systems, named type I to VI (T1SS to T6SS) identified in Gram(−) bacteria [1], T6SS is the most recently identified system and is widespread in *Proteobacteria* [2]–[5]. Many T6SSs of pathogenic bacteria are induced inside the host or in response to host signals, which suggests their functions during bacterium–host interactions [6]. Numerous studies in various bacteria further suggested the diversified functions of T6SS, including survival within the host, escape from host predation, killing of eukaryotic or bacterial host cells, biofilm formation, stress response, and quorum sensing [7]–[13]. Growing evidence from structural and functional studies further reveals that T6SS may assemble into a bacteriophage tail-like structure to deliver effectors into the recipient cells [5], [14].

The diverse functions of T6SS are reflected by its regulation by multiple mechanisms. T6SS is regulated at epigenetic, transcriptional, posttranscriptional, and posttranslational levels [3], [15]–[17]. In enteroaggregative *Escherichia coli*, the *sci1* T6SS gene cluster is under the control of an epigenetic switch regulated by iron availability through Fur- and Dam-dependent methylation [15]. Several T6SSs are transcriptionally regulated by various two-component systems, transcription factors, quorum sensing, alternative sigma factor 54, and histone-like proteins [18]–[22]. The regulation of T6SS gene cluster expression by a two-component system has been reported for several T6SSs, including those from *Burkholderia mallei* by VirA/VirG, *Edwarsiella tarda* by EsrA/EsrB, and *Salmonella enterica* by SsrA/SsrB [20], [23], [24]. Posttranscriptional or translational control was revealed with the RNA binding protein RsmA, which acts as a translation repressor of the mRNA level of *Pseudomonas aeruginosa* HSI-1 T6SS [25]. In *P. aeruginosa*, HSI-1 T6SS is posttranslationally regulated by serine/threonine kinase PpkA and the cognate phosphatase PppA via threonine phosphorylation on a forkhead-associated protein, Fha1 [26]. These multiple regulatory cascades suggest that the versatile control of T6SS is critical for its function in response to specific signals.

*Agrobaterium tumefaciens* is a soil bacterium causing crown gall disease in a wide range of plants. It integrates transferred DNA (T-DNA) from the tumor-inducing plasmid into the host genome [27]–[29]. When *A. tumefaciens* encounters signals such as acidity, monosaccharides, and phenolic compounds released from plant wound sites, the VirA/VirG two-component system, in cooperation with periplasmic sugar binding protein ChvE, is activated to induce the expression of virulence (*vir*) genes to direct the transfer of T-DNA into host cells [28], [30]. T4SS, comprising 11 VirB proteins and VirD4 forming a transmembrane multi-protein complex, is responsible for the transfer of T-DNA and effector proteins from bacteria into host plant cells [31]. In addition to VirA/VirG, which is responsible for the expression of *vir* genes encoded by tumor-inducing plasmid, the chromosome-encoded ChvG/ChvI two-component system is responsible for the expression of acidity-inducible genes, including *aopB*, encoding an outer membrane protein; *pckA*, encoding phosphoenolpyruvate carboxykinase; and *virG* [32]–[36]. ChvG is a typical transmembrane sensor kinase that contains a large periplasmic domain located between 2 transmembrane domains and a conserved histidine kinase domain in the C-terminal cytoplasmic region [32]. The ChvG/ChvI two-component system has an essential role in tumor formation and bacterial growth under acidic conditions and in membrane integrity [33], [37].

The ChvG/ChvI two-component system is highly conserved in *α-Proteobacteria* [32], [37]–[40]. In the plant symbiont *Sinorhizobium meliloti*, the ChvG ortholog ExoS is the sensor kinase and with the response regulator ChvI, functions as the positive regulator for synthesis of exopolysaccharide succinoglycan, promoting biofilm formation and motility [41]. In *S. meliloti*, ExoR regulates its own expression through ExoS/ChvI [42] and functions as a periplasmic regulator inhibiting ExoS/ChvI signaling by physical interaction with ExoS [41], [43]. Recent study also revealed that periplasmic ExoR is targeted for proteolysis [44]; however, whether and how ExoR perceives signals and the molecular mechanisms underlying how ExoR regulates ExoS(ChvG)/ChvI activity remain unknown. Interestingly, ExoR can also function independently of ChvG/ChvI in repressing succinoglycan biosynthesis and promoting biofilm formation and motility in *A. tumefaciens* [45].

How T6SS is regulated in *A. tumefaciens* remains largely unknown. Previously, we found the secretion of the T6SS hallmark protein hemolysin-coregulated protein (Hcp) in *A. tumefaciens* grown under various conditions, including nutrient-rich or minimal medium at low (19°C) or room temperature [46]. Interestingly, transcriptome assays revealed that the expression of several T6SS genes encoded by the *imp* operon is higher under acidic than neutral minimal medium conditions [36]. However, whether the acid-induced *imp* gene expression is responsible for activation of T6SS secretion and the regulatory mechanisms underlying T6SS expression and activity are unknown.

In this study, we aimed to investigate whether the expression and secretion of *A. tumefaciens* T6SS is regulated by plant-derived signals and if so, the underlying regulatory mechanism. T6SS-mediated Hcp secretion was almost silent with *A. tumefaciens* grown in neutral minimal medium but was induced by acidity. Further molecular analysis revealed that T6SS is activated by the ChvG/ChvI two-component system, with the sensor kinase ChvG negatively regulated by the periplasmic repressor ExoR. Importantly, we provide the first evidence that acidity induces ExoR degradation, which then may derepress ChvG to activate T6SS through a ChvI response regulator in a phosphorylation-dependent manner. This activation of T6SS by an acidic signal present in plant wound sites and apoplasts (intercellular space) suggests its potential role during *Agrobacterium* infection or replication near or inside plants.

## Results

### Acidity activates T6SS expression and secretion

**Figure 1:**
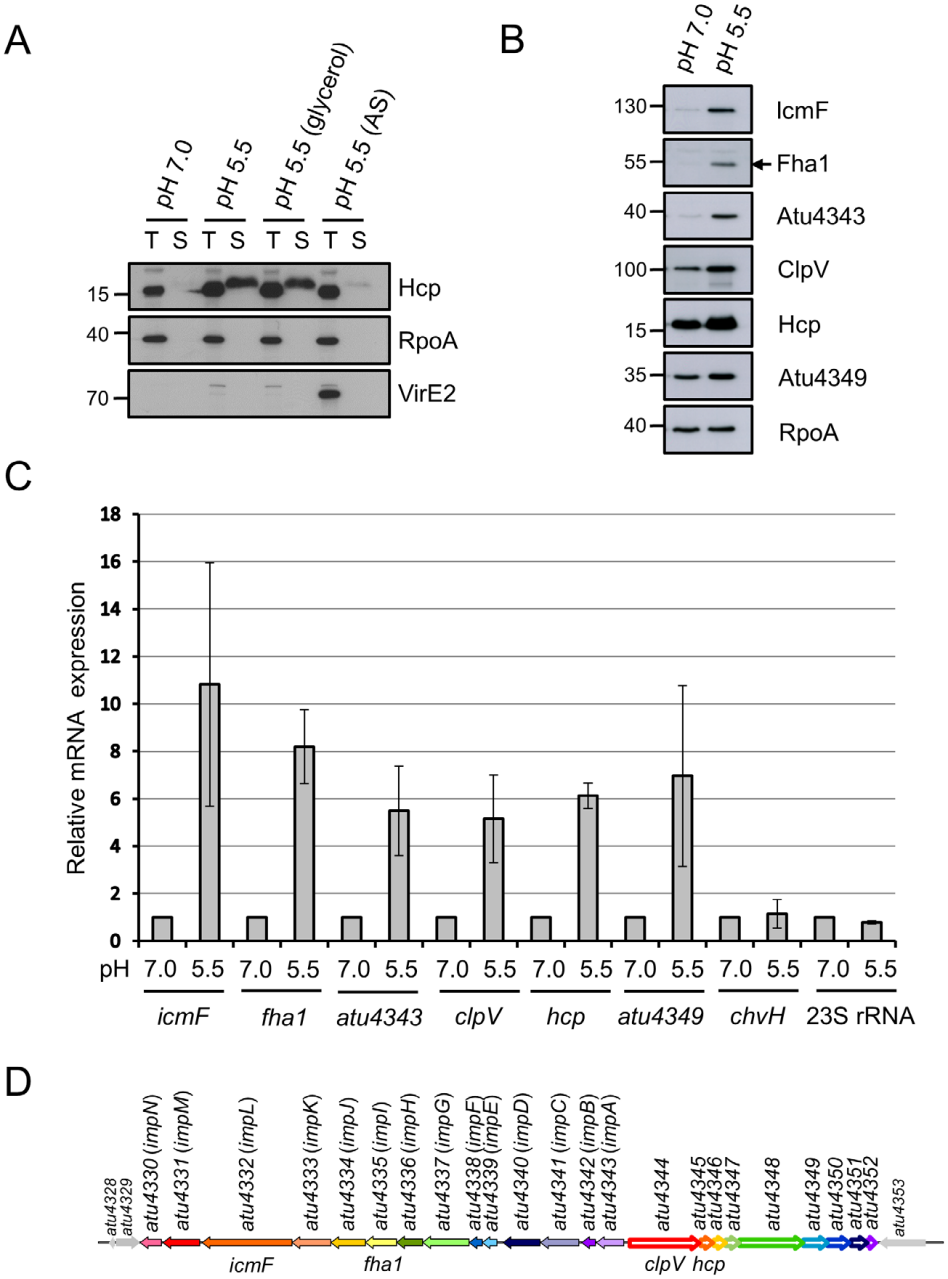
T6SS expression and Hcp secretion are concomitantly upregulated by acidity. *A. tumefaciens* wild-type C58 strain grown in AB-MES (pH 7.0 or pH 5.5) in the presence of substituting carbon source (glycerol) or acetosyringone (AS) at 25°C for 6 h were analyzed for T6SS expression and Hcp secretion. (**A**) Total (T) and secreted (S) proteins were analyzed by western blot analysis with antibodies against Hcp, RpoA, and VirE2. (**B**) Total proteins were analyzed by western blot analysis with antibodies against C-IcmF, Fha1 (filled arrow), Atu4343, ClpV, Hcp, Atu4349, and RpoA. The non-secreted protein RpoA was an internal control. The positions of molecular mass markers (in kDa) are indicated on the left. (**C**) Quantitative RT-PCR (qRT-PCR) analysis of mRNA levels of indicated genes. Data are mean ± SD of 2 or 3 biological replicates, each of which contains 3 technical replicates. (**D**) Genetic organization of the *A. tumefaciens* T6SS gene cluster. The T6SS gene cluster consists of 2 operons with divergent orientation. The *imp* operon encoding 14 proteins is indicated by filled colored arrows and the *hcp* operon encoding 9 proteins by open colored arrows. The locus number and published/annotated gene designation are indicated above and below each gene, respectively.

Our previous secretome analysis revealed abundant secretion of the T6SS hallmark protein Hcp from *A. tumefaciens* grown in acidic minimal medium (AB-MES, pH 5.5) [46]. Thus, we have been routinely using this growth condition to monitor T6SS activity in *A. tumefaciens* by Hcp secretion assay [47], [48]. However, the signals responsible for activating T6SS for Hcp secretion remain unclear. Because *A. tumefaciens* can sense plant-derived signals, including acidity, monosaccharides, and phenolic compounds, which are critical components in the acidic minimal medium (AB-MES, pH 5.5) for *vir* gene expression, we first tested whether any of these 3 signals plays a role in regulating T6SS activity. *A. tumefaciens* wild-type strain C58 cells cultured overnight in AB-MES (pH 7.0) was sub-cultured in neutral (pH 7.0) or acidic (pH 5.5) AB-MES minimal medium with or without monosaccharides or acetosyringone (AS) phenolics for 6 h. Secretion of Hcp was abundant from *A. tumefaciens* cells grown in acidic minimal medium (pH 5.5) (Figure 1A), with barely detected secretion from cells grown in neutral minimal medium (pH 7.0). Sugar or carbon source did not seem to regulate Hcp secretion because secretion did not differ in cultures supplemented with carbon sources such as glucose, sucrose, cellobiose, or glycerol (Figure 1A and Figure S1A). Intriguingly, the addition of AS in acidic minimal medium, which induces *vir* gene expression, as evidenced by VirE2 expression, significantly attenuated Hcp secretion as compared with no AS (Figure 1A). The non-secreted protein RNA polymerase subunit A (RpoA) was an internal control. DMSO, used to dissolve AS, did not reduce Hcp secretion grown under acid-inducing conditions (Figure S1B). Therefore, T6SS-mediated Hcp secretion was almost silent in *A. tumefaciens* grown in neutral minimal medium and was activated when the acidic signal was sensed. The attenuation of Hcp secretion in AS-induced acidic medium, which can induce the expression of *vir* genes, implied a complex regulatory network during *Agrobacterium*–plant interactions. Here, we investigated the regulatory mechanism underlying the acid-induced expression and secretion of T6SS.

Systematic mutagenesis analysis of the T6SS locus from *Edwardsiella tarda* and *Vibrio cholera*, along with other studies, revealed about a dozen conserved components essential for mediating T6SS secretion [5], [49], [50]. In *A. tumefaciens*, the T6SS gene cluster comprises 2 divergently transcribed operons: *imp*, encoding 14 genes (*atu4343* to *atu4330*); and *hcp*, encoding 9 genes (*atu4344* to *atu4352*) (Figure 1D). To examine the regulation of acid-induced Hcp secretion, quantitative RT-PCR (qRT-PCR) and western blot analyses revealed greatly upregulated expression of 3 selected genes encoded by the *imp* operon (*icmF, fha1, atu4343*) and 3 by the *hcp* operon (*clpV, hcp, atu4349*) with acidity (AB-MES, pH 5.5) (Figure 1C). As controls, 23S rRNA and *chvH* genes, known not to respond to pH change [32], showed similar mRNA levels with both acidic and neutral medium. The proteins encoded by the *imp* operon (IcmF, Fha1, Atu4343) were barely detected when grown in neutral minimal medium, whereas those encoded by the *hcp* operon (ClpV, Hcp, Atu4349) were expressed at substantial levels under this growth condition (Figure 1B). The levels of proteins encoded by the *imp* operon were markedly induced by acidity, whereas levels of proteins encoded by the *hcp* operon were only modestly higher with acidic than neutral medium (Figure 1B). Therefore, T6SS secretion was activated by acid-induced expression of T6SS genes, especially those encoded by the *imp* operon. This pH-regulated T6SS expression and secretion were also observed in other *A. tumefaciens* strains such as Ach5 and 1D1609 [51], [52] (Figure S2A and S2B), which suggests that this may be a common regulatory mechanism in *A. tumefaciens*.

### ExoR and ChvG/ChvI two-component system coordinate to regulate T6SS expression and Hcp secretion

**Figure 2:**
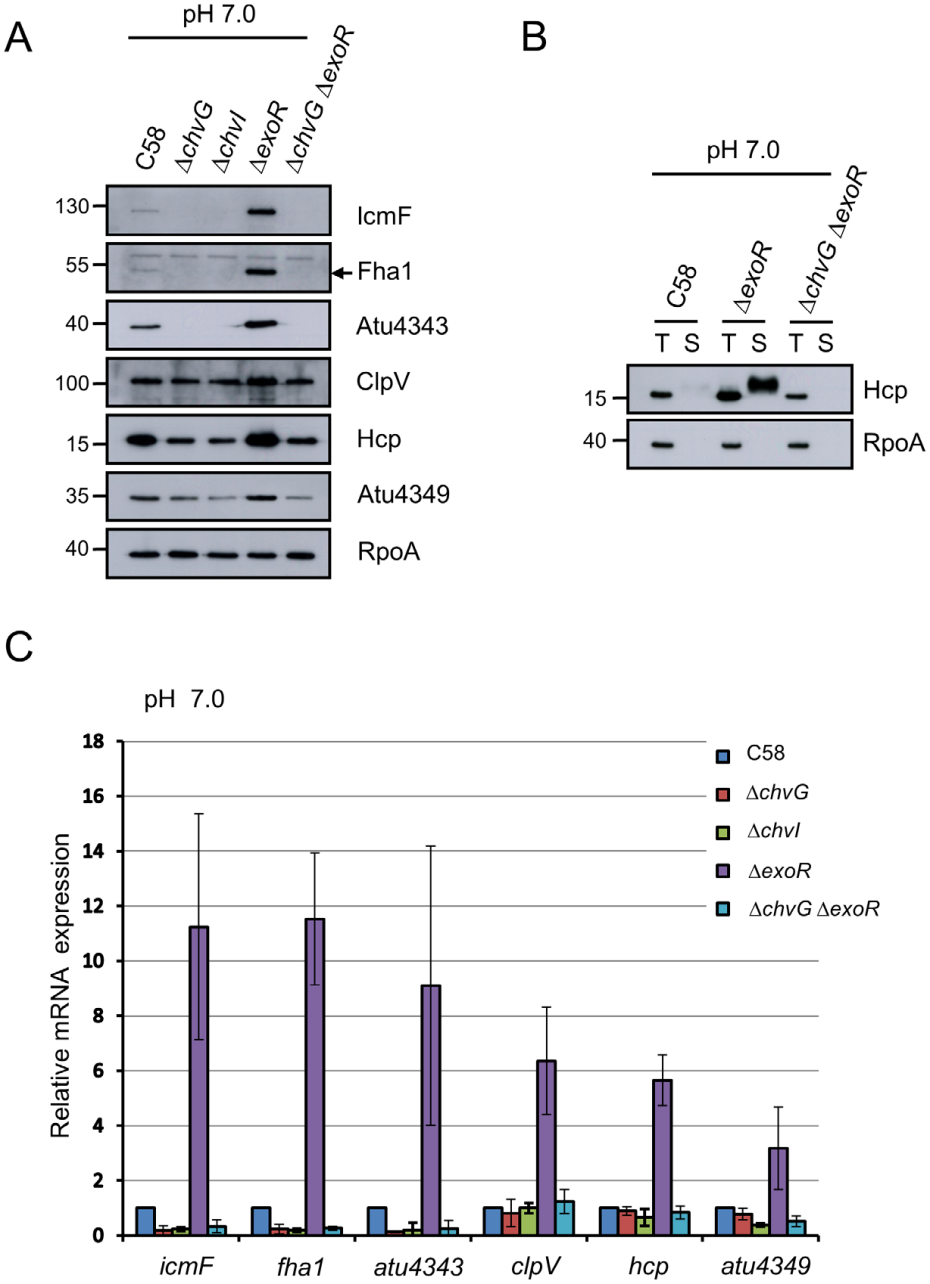
T6SS expression and Hcp secretion are positively regulated by ChvG/ChvI and negatively regulated by ExoR. *A. tumefaciens* wild-type strain C58, *ΔchvG*, *ΔchvI*, *ΔexoR*, and *ΔchvG ΔexoR* mutant strains grown in AB-MES (pH 7.0) at 25°C for 6 h were analyzed for T6SS expression and secretion. (**A**) Total proteins were analyzed by western blot analysis with antibodies against C-IcmF, Fha1 (filled arrow), Atu4343, ClpV, Hcp, Atu4349, and RpoA. (**B**) Total (T) and secreted (S) proteins were analyzed by western blot analysis with antibodies against Hcp and RpoA. The non-secreted protein RpoA was an internal control. The positions of molecular mass markers (in kDa) are indicated on the left. (**C**) qRT-PCR analysis of mRNA levels of indicated genes. Data are mean ± SD of 2 biological replicates, each of which contains 3 technical replicates.

The finding that acidic pH is the key to trigger T6SS gene expression and thus Hcp secretion prompted us to search for pH-responsive regulatory genes in *A. tumefaciens*. The ChvG/ChvI two-component system functions to regulate certain acidity-inducible genes, including the system itself, and may serve as a global pH sensor in *A. tumefaciens* [32], so it may be a candidate for testing the regulatory role in T6SS. We first generated *ΔchvG* and *ΔchvI* in-frame deletion mutants for the two-component system and examined the effects on T6SS expression and Hcp secretion. Because *ΔchvG* and *ΔchvI* are sensitive to a nutrient-rich or acidic environment [37], both mutants were grown and maintained in neutral minimal medium (AB-MES, pH 7.0). The proteins encoded by the *imp* or *hcp* operon showed different basal levels in neutral minimal medium (AB-MES, pH 7.0), and their protein levels were all further reduced with *chvG* or *chvI* deletion (Figure 2A). However, as compared with *chvG/chvI* likely being essential for the expression of the *imp* operon, neither *chvG* nor *chvI* were absolutely required for *hcp* operon expression, as determined by western blot and qRT-PCR analyses (Figure 2A and 2C).

Because ExoS(ChvG)/ChvI signaling is negatively regulated by ExoR in *S. meliloti* [43], we investigated whether ExoR functions upstream of ChvG/ChvI in regulating T6SS in *A. tumefaciens*. Lack of *exoR* enhanced the expression of both *imp* and *hcp* operons (Figure 2A and 2C), which indicates that *exoR* negatively regulates T6SS expression in *A. tumefaciens*. Deletion of *chvG* in the *ΔexoR* mutant background abrogated the induced expression of *imp* and *hcp* operons (Figure 2A and 2C), which indicates that *chvG* is epistatic to *exoR* in regulating T6SS. Hcp secretion was increased in *ΔexoR*, and this enhancement was abolished in the *ΔchvG ΔexoR* mutant under this growth condition (AB-MES, pH 7.0) (Figure 2B). Therefore, T6SS is regulated positively by *chvG/chvI* and negatively by *exoR*, which likely functions upstream of *chvG* sensor kinase. Of note, *imp* expression is tightly regulated by *exoR* and *chvG/chvI*, which are not absolutely required for the expression of the *hcp* operon. These results also suggest that acid-induced T6SS secretion is mainly controlled by the expression of *imp*-encoding proteins constituting the T6S machinery. The basal expression of the *hcp* operon in the absence of *chvG* or *chvI* further suggested the existence of an additional regulatory pathway for *hcp* operon expression.

### Phospho-mimic but not unphosphorylated ChvI can bind the T6SS promoter region and activate T6SS

**Figure 3:**
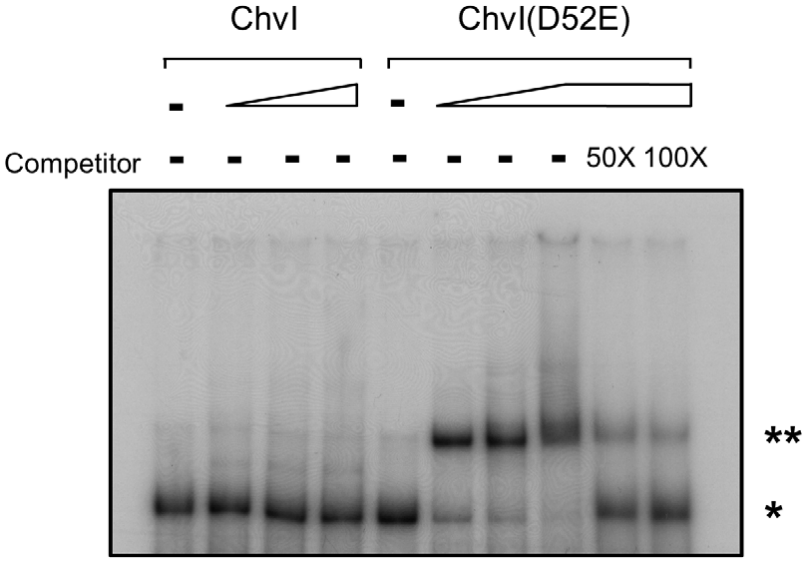
Phospho-mimic ChvI(D52E) binding to T6SS regulatory region. The ^32^P-labeled DNA fragment of 230-bp intergenic region between the divergent *imp* and *hcp* operons was incubated without or with increasing amounts of purified recombinant wild-type ChvI or ChvI(D52E) proteins (11, 33, and 100 ng). The binding was completed by the addition of 50- and 100-fold of excess unlabeled probe as a specific competitor. The protein–DNA complexes were resolved on 5%TBE acrylamide gel. The ChvI–DNA complex and free probe are indicated by * and ** respectively.

To relay signals via a two-component system, a sensor kinase is activated by the input signal and phosphorylates the cognate response regulator, which then exerts its function by regulating its target gene [53]. Thus, we wondered whether ChvI, as a response regulator, binds directly to the promoters of the divergent *imp* and *hcp* operons. We used electrophoretic mobility shift assay (EMSA) and incubated the purified His-ChvI recombinant protein with a 230-bp DNA fragment derived from the intergenic region of the *imp* and *hcp* operons but detected no binding activity (Figure 3). Because the phosphorylated state of the response regulator could modulate its DNA binding activity, we then expressed and purified the ChvI variant by replacing the conserved aspartic acid phosphorylation site with glutamic acid (D52E), a phospho-mimic variant previously shown to be constitutively active in other systems [54], [55]. EMSA revealed strong binding of the phospho-mimic variant to the probe, and the shifted complex was dissociated when challenged with the unlabeled specific DNA competitor (Figure 3). As a control, ChvI(D52E) did not bind to an unrelated DNA fragment derived from the *A. tumefaciens* genome (Figure S3). Thus, ChvI may be the response regulator directly regulating T6SS, and the phosphorylated state of ChvI is crucial for its direct binding to the intergenic promoter region between both operons.

**Figure 4:**
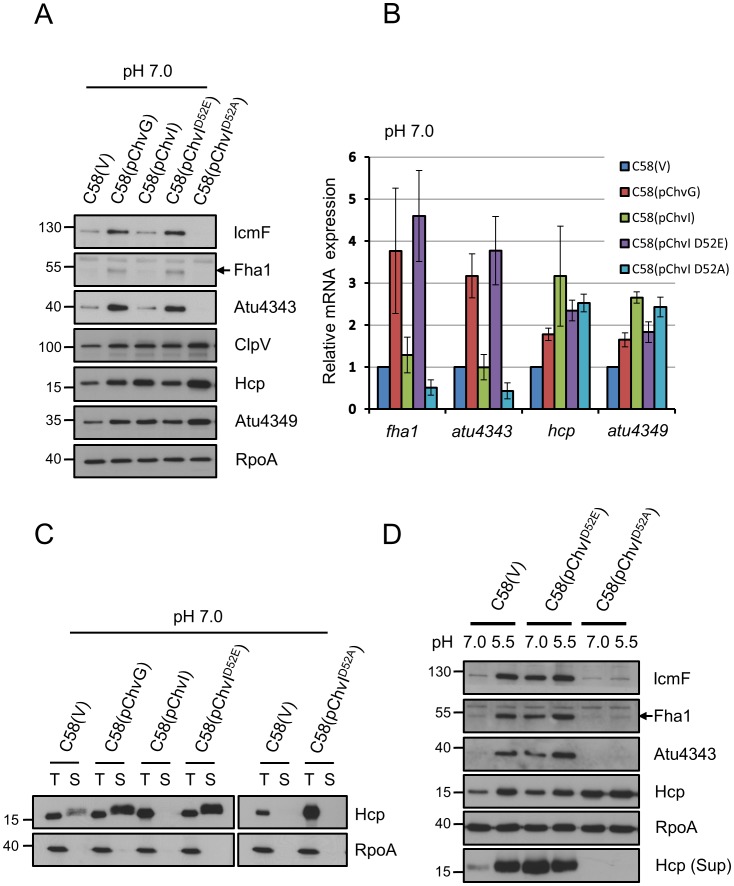
Phosphorylated status of ChvI regulates T6SS expression and Hcp secretion. *A. tumefaciens* wild-type C58 strain containing the empty vector (V) or one of the plasmids expressing ChvG, ChvI, ChvI(D52E) or ChvI(D52A) grown in AB-MES (pH 7.0 or 5.5) at 25°C for 6 h was analyzed for T6SS expression and Hcp secretion. (**A**) Total proteins were analyzed by western blot analysis with antibodies against C-IcmF, Fha1 (filled arrow), Atu4343, ClpV, Hcp, Atu4349, and RpoA. (**B**) qRT-PCR analysis of mRNA levels of *fha1*, *atu4343*, *hcp*, and *atu4349*. Data are mean ± SD of 2 biological replicates, each of which contains 3 technical replicates. (**C**) Total (T) and secreted (S) proteins were analyzed by western blot analysis with antibodies against Hcp and RpoA. (**D**) Total and secreted (Sup) proteins were analyzed by western blot analysis with antibodies against C-IcmF, Fha1 (filled arrow), Atu4343, Hcp, and RpoA. RpoA was an internal control. The positions of molecular mass markers (in kDa) are indicated on the left.

To determine whether the binding of phospho-mimic ChvI(D52E) to the T6SS promoter region *in vitro* was biologically significant *in vivo*, we overexpressed ChvI(D52E) in *A. tumefaciens* wild-type C58 to determine whether this phospho-mimic ChvI was sufficient to trigger T6SS expression and secretion in neutral medium, the secretion-repression condition. In parallel, we overexpressed the sensor kinase ChvG and wild-type ChvI in C58 as controls. Both the mRNA and protein levels of the analyzed *imp* genes were significantly induced with ChvG and ChvI(D52E) overexpression as compared with vector expression alone (Figure 4A and 4B). In contrast, overexpression of wild-type ChvI did not elevate the mRNA and protein levels of genes encoded by the *imp* operon. Moreover, overexpression of ChvI(D52A), with inactivation of the ChvI phosphorylation site, further reduced *imp* gene expression at both mRNA and protein levels.

Interestingly, the expression of the *hcp* operon was regulated differently from the *imp* operon with the overexpression strains. As expected, *hcp* operon expression was increased at both mRNA and protein levels with ChvG and ChvI(D52E) overexpression (Figure 4A and 4B, Figure S4). Surprisingly, ChvI and ChvI(D52A) overexpression increased the *hcp* operon expression at both mRNA and protein levels (Figure 4A and 4B). Hcp secretion was activated by overexpression of ChvG and the phospho-mimic ChvI(D52E) but not wild-type ChvI or ChvI(D52A) (Figure 4C), which supports Hcp secretion being controlled by the expression of the *imp* operon. Moreover, phospho-mimic ChvI(D52E) and phospho-inactive ChvI(D52A) were insensitive to the acidity in regulating T6SS, with evidence that ChvI(D52E) was constitutively active and ChvI(D52A) defective in T6SS expression and secretion in both neutral and acidic medium (Figure 4D). These data suggest that activation of *imp* operon expression requires phosphorylated ChvI, but phosphorylated or unphosphorylated ChvI can upregulate the *hcp* operon.

### Expression analysis of *chvG* and *exoR* in response to acidity

**Figure 5:**
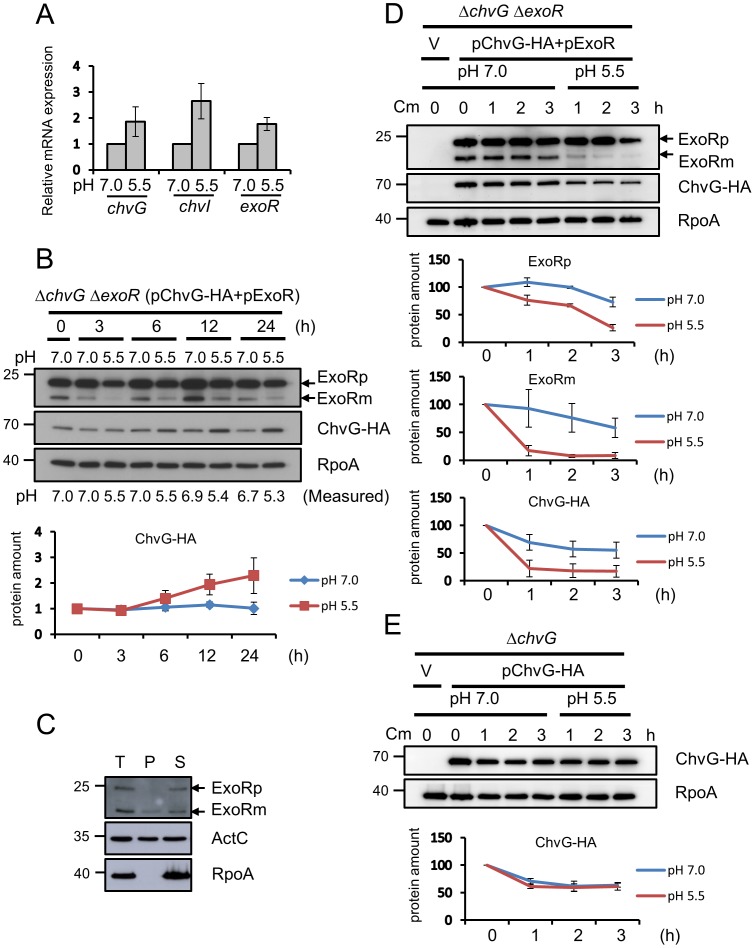
Effect of acidity on expression and protein stability of ChvG and ExoR. (**A**) qRT-PCR analysis of mRNA levels of *chvG*, *chvI*, and *exoR* for wild-type C58 strain grown in AB-MES (pH 7.0 or pH 5.5) at 25°C for 6 h. Data are mean ± SD of 2 biological replicates, each of which contains 3 technical replicates. (**B**) Total proteins isolated from *ΔchvG ΔexoR* expressing pChvG-HA and pExoR grown in AB-MES (pH 7.0 or pH 5.5) at 25°C for the indicated times underwent western blot analysis with antibodies against ExoR (filled arrow), HA (for detecting ChvG-HA), and RpoA. The protein level of ChvG-HA was quantified and normalized to the level of endogenous RpoA. The amount of ChvG-HA at 0 h was set to 1. Data are mean ± SD of 2 biological replicates. (**C**) Equal volumes of total proteins (T), periplasmic fraction (P), and spheroplast pellets (S) isolated from *ΔexoR*(pExoR) grown in AB-MES (pH 7.0) at 25°C for 6 h were analyzed by western blot analysis with antibodies against ExoR, ActC, and RpoA. ActC was a control of periplasmic protein. (**D**) Total proteins isolated from *ΔchvG ΔexoR* containing the empty vector (V) or one of the plasmids expressing pChvG-HA and pExoR grown in AB-MES (pH 7.0) or AB-MES (pH 5.5) in the presence of chloramphenicol at 25°C for the indicated times underwent western blot analysis with antibodies against ExoR (filled arrow), HA (for detecting ChvG-HA), and RpoA. The protein level of precursor ExoR (ExoRp) and mature ExoR (ExoRm), and ChvG-HA were quantified with the level present at 0 h set to 100%. Data are mean ± SD of 3 biological replicates. (**E**) Total proteins isolated from *ΔchvG* expressing ChvG-HA grown in AB-MES (pH 7.0 or pH 5.5) in the presence of chloramphenicol at 25°C for the indicated times underwent western blot analysis with antibody HA (for detecting ChvG-HA). The protein level of ChvG-HA was quantified with the level present at 0 h was set to 100%. Data are mean ± SD of 2 biological replicates.

The evidence that c*hvG* is epistatic to *exoR* in regulating T6SS (Figure 2) suggested that *exoR* functions upstream of *chvG/chvI* to abrogate ChvG/ChvI-induced T6SS activity. Because overexpression of ChvG in the presence of *exoR* could activate T6SS in *A. tumefaciens* grown in neutral medium, we hypothesized that acid-induced T6SS expression and secretion are activated by increased ChvG protein level, which is negatively regulated by ExoR in neutral medium. Thus, we first examined whether the expression of *chvG, chvI*, and *exoR* were regulated by acidity. As expected, the mRNA levels of both *chvG* and *chvI* were higher in acidic than neutral medium (Figure 5A), which agreed with previous findings for acid-induced *chvI-chvG* autoregulation [36]. Interestingly, *exoR* mRNA levels were slightly upregulated by acidity (Figure 5A).

**Figure 6:**
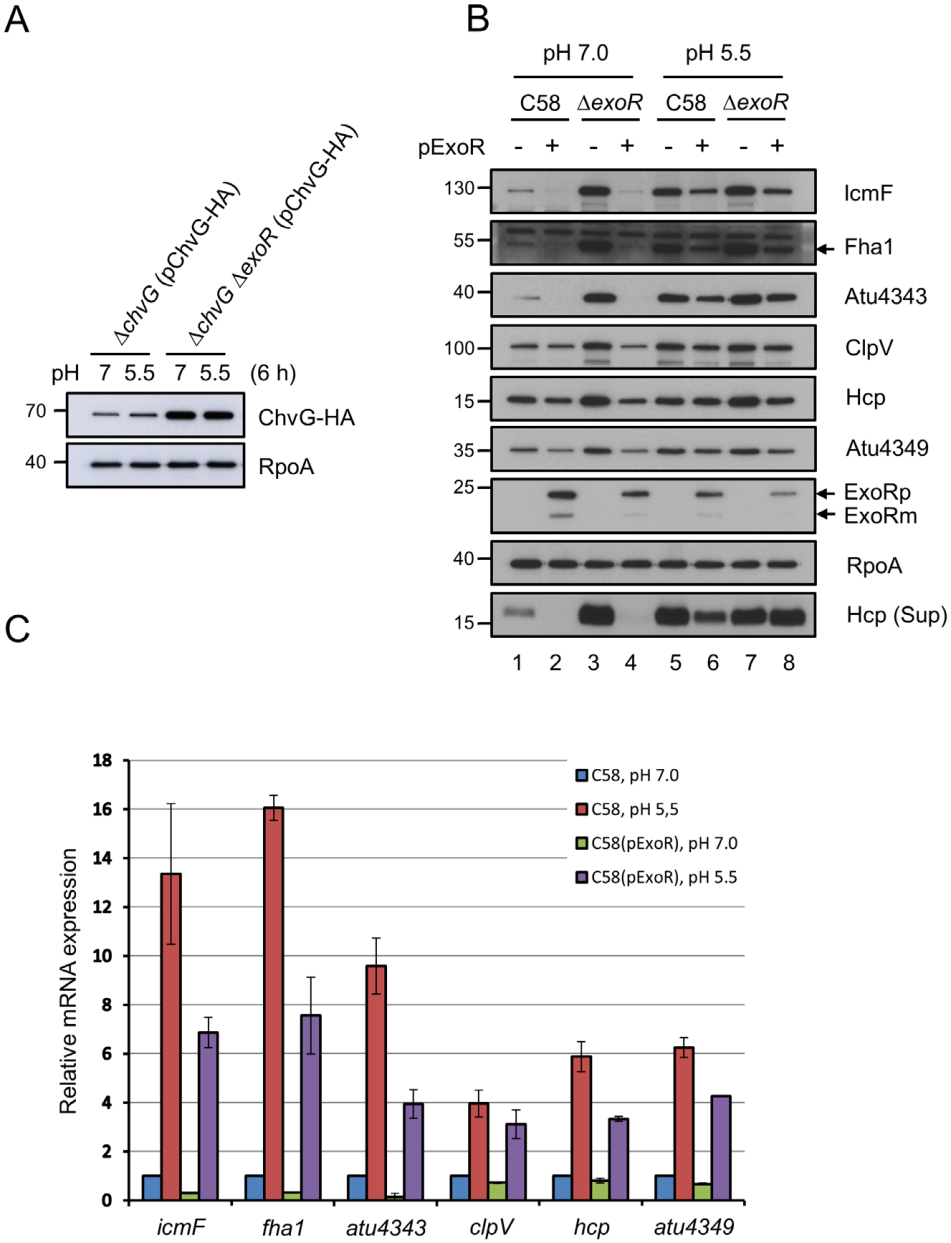
Effect of overexpressed ExoR in ChvG levels and T6SS expression/secretion in both neutral and acidic medium. (**A**) Western blot analysis of ChvG-HA protein level in total proteins isolated from *A. tumefaciens ΔchvG* or *ΔchvGΔexoR* strains containing pChvG-HA grown in AB-MES (pH 7.0 or pH 5.5) at 25°C for 6 h. (**B**) Total and secreted (Sup) proteins isolated from *A. tumefaciens* wild-type C58 and *ΔexoR* mutant strain expressing ExoR grown in AB-MES (pH 7.0 or pH 5.5) at 25°C for 6 h were analyzed by western blot analysis with antibodies against C-IcmF, Fha1 (filled arrow), Atu4343, ClpV, Hcp, Atu4349, ExoR (filled arrow), and RpoA. RpoA was an internal control. The lane numbers are indicated at the bottom and the positions of molecular mass markers (in kDa) are indicated on the left. (**C**) qRT-PCR analysis of *icmF*, *fha1*, *atu4343*, *clpV*, *hcp*, and *atu4349* mRNA level isolated from wild-type and *ΔexoR* strains expressing ExoR grown in AB-MES (pH 7.0 or pH 5.5) at 25°C for 6 h. Data are mean ± SD of 2 biological replicates, each of which contains 3 technical replicates.

We next determined the protein levels of ChvG and ExoR in *A. tumefaciens* grown under both neutral and acidic conditions. Because of the lack of ChvG-specific antibody and inability to detect endogenous ExoR protein in wild-type C58 (Figure 6B), we used heterologous promoters to overexpress ExoR and ChvG in *A. tumefaciens* under both neutral and acidic conditions. ChvG tagged with hemagglutinin (HA) at the C terminus was driven by a constitutively active *lac* promoter from pRL622 (P*lac*-ChvG-HA), and ExoR was expressed by an isopropyl-beta-D-thiogalactoside (IPTG)-inducible *trc* promoter from pTrc200 (P*trc*-ExoR). The ectopic expression of ExoR and ChvG also allowed us to monitor their protein expression and stability at posttranscriptional levels. The AB-MES (pH 7.0) overnight-grown bacterial culture was subcultured in neutral (pH 7.0) or acidic (pH 5.5) AB-MES medium for 3, 6, 12, and 24 h and collected for western blot analysis. Both neutral and acidic medium maintained the pH over this time because we detected only a slight decrease of pH after 12- to 24-h growth (Figure 5B). ChvG protein level was increased steadily in acidic culture, with a pronounced increase at 12 and 24 h, whereas ChvG protein level remained at lower levels in neutral medium (Figure 5B). For ExoR, which contains an N-terminus signal sequence targeting ExoR to periplasm in *S. meliloti* [41], we detected 2 protein bands specifically recognized by the antibody against *S. meliloti* ExoR (Figure 5B). Biochemical fractionation results indicated that the upper band represents the cytoplasmic ExoR precursor (ExoRp), which contains the unprocessed signal sequence, whereas the lower band is the mature periplasmic ExoR (ExoRm), with removal of the signal peptide (Figure 5C). ExoR protein levels were decreased at 3 h after shifting to acidic medium as compared with neutral medium (Figure 5B). Although ExoR continued to be synthesized in neutral or acidic medium, as revealed by increased protein levels up to 12 h, levels of both ExoRp and periplasmic ExoRm were lower in acidic medium.

### Stability analysis of ExoR and ChvG proteins in response to acidity

Next, we determined whether the low levels of periplasmic ExoRm under the acidic condition is caused by the instability of ExoRm when *A. tumefaciens* senses acidity. Thus, we traced both ExoR and ChvG protein stability by western blot analysis in the P*lac*-ChvG-HA and P*trc*-ExoR overexpression strain with protein synthesis inhibited by chloramphenicol. Periplasmic ExoRm protein level was rapidly decreased in acidic medium as compared with neutral medium, with less effect on stability of unprocessed ExoRp with neutral or acidic medium (Figure 5D). Interestingly, ChvG-HA was also less stable in acidic than neutral medium with overexpressed ExoR (Figure 5D). However, ChvG-HA was more stable, with similar stability in both acidic and neutral medium with endogenous *exoR* (Figure 5E). The differential stability of ChvG with or without detectable ExoR in response to acidity suggested a negative role of ExoR in ChvG protein stability in the acidic environment. However, ChvG retains similar protein stability independent of ExoR protein levels when grown in neutral medium.

### ChvG protein levels and T6SS expression and secretion are negatively regulated by *exoR*

Next, we determined whether the higher ChvG protein levels in acidic than neutral medium (Figure 5B) are regulated by *exoR*. To test this possibility, P*lac*-ChvG-HA was expressed in both *ΔchvG* and *ΔchvGΔexoR* mutants to monitor whether increased ChvG protein level is regulated by *exoR* at the posttranscriptional level. The level of ChvG protein was slightly higher in acidic than neutral medium at 6 h with *exoR*; however, the level of ChvG-HA was greatly increased without *exoR* and comparable in neutral and acidic medium (Figure 6A). These data suggest that *exoR* negatively regulates ChvG protein levels in both acidic and neutral medium. Importantly, in contrast to acid-induced T6SS expression in C58 (Figure 6B, lane 1 vs. lane 5), the levels of proteins encoded by *imp* and *hcp* operons were not further increased in *ΔexoR* in response to acidity (Figure 6B, lane 3 vs. lane 7). This result is consistent with comparable ChvG protein levels in *ΔexoR* grown in neutral or acidic minimal medium (Figure 6A). Furthermore, the acid-induced T6SS expression and secretion were largely compromised when ExoR was overexpressed (Figure 6B, lane 5 vs. lane 6; and 6C). Because *exoR* mRNA levels were not reduced (Figure 5A) but periplasmic ExoRm was rapidly degraded in response to acidity (Figure 5D), the acidity-triggered ExoRm degradation may contribute to the elevated ChvG protein abundance and thereby lead to T6SS activation under acidic conditions.

### ExoR–ChvG interaction regulates ChvG protein level and T6SS expression/secretion

The inverse association of ExoR and ChvG protein level, together with the epistasis of *chvG* to *exoR* prompted us to investigate the mode of action of ExoR in repressing ChvG/ChvI signaling. In *S. meliloti*, periplasmic ExoR physically interacts with ExoS (ChvG ortholog), and this interaction is critical for inhibiting ExoS/ChvI signaling [43]. Thus, ExoR may negatively regulate T6SS activity by physical interaction with ChvG in *A. tumefaciens* grown under neutral conditions. When the acidic signal is sensed, the degradation of ExoRm may thereby allow the inner-membrane–associated ChvG sensor kinase to activate the ChvI response regulator and induce T6SS expression and secretion activity.

**Figure 7:**
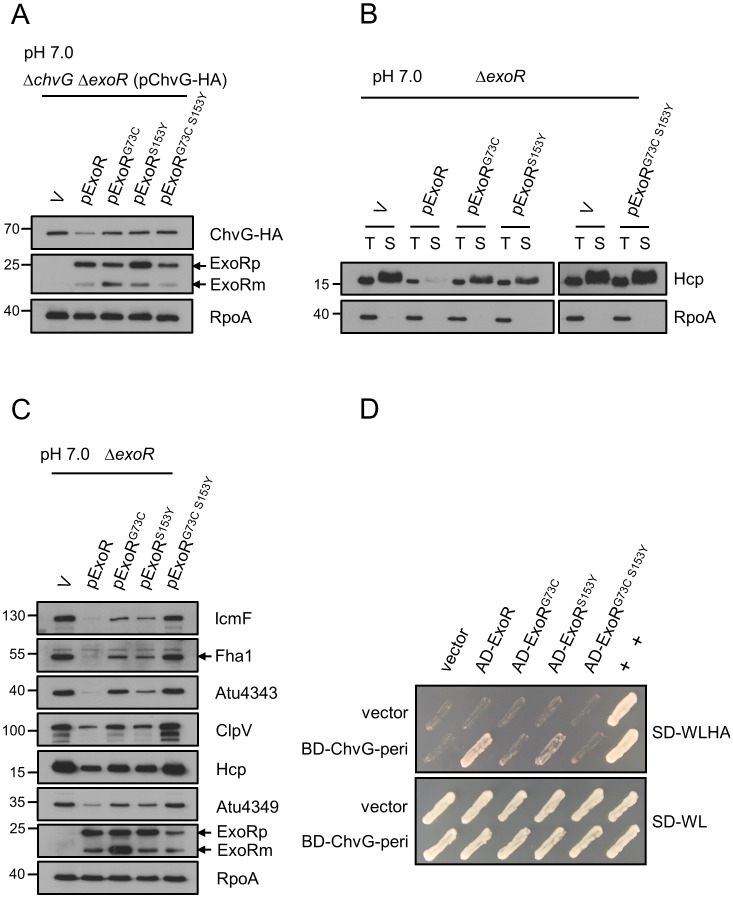
Effect of ExoR and ChvG associations on T6SS expression and Hcp secretion. *A. tumefaciens* strain *ΔchvG ΔexoR*(pChvG-HA) containing the empty vector (V) or one of the plasmids expressing wild-type ExoR, ExoR(G73C), ExoR(S153Y), or ExoR(G73C S153Y) grown in AB-MES (pH 7.0) at 25°C for 6 h were analyzed for T6SS expression and Hcp secretion. (**A**) Total proteins were analyzed by western blot analysis with antibodies against HA (for detecting ChvG-HA), ExoR (filled arrow), and RpoA. (**B**) Total (T) and secreted (S) proteins were analyzed by western blot analysis with antibodies against Hcp and RpoA. (**C**) Total proteins were analyzed by western blot analysis to detect T6SS proteins with antibodies against C-IcmF, Fha1 (filled arrow), Atu4343, ClpV, Hcp, Atu4349, ExoR, and RpoA. The non-secreted protein RpoA was an internal control. The positions of molecular mass markers (in kDa) are indicated on the left. (**D**) Yeast two-hybrid protein–protein interaction results with the periplasmic domain of ChvG and various ExoR proteins. SD-WL medium (SD minimal medium lacking Trp and Leu) was used for selection of plasmids. SD-WLHA medium (SD minimal medium lacking Trp, Leu, His, and Ade) was used for auxotrophic selection of bait and prey protein interactions. The positive interaction was determined by growth on SD-WLHA medium at 30°C for at least 2 days. The positive control (+) showing interactions of SV40 large T-antigen and murine p53 and negative control (vector) are indicated.

Thus, we aligned the amino acid sequences of ExoR encoded by *A. tumefaciens* and *S. meliloti* and identified the putative amino acid residues in *A. tumefaciens* ExoR that may be critical for interaction with ChvG (Figure S5). ExoR contains a conserved N-terminal signal peptide and tetratricopeptide repeat (TPR)/Sel1-like domains, which are implicated in protein–protein interactions [56]. We generated the *exoR* mutants encoding ExoR variants with amino acid substitution mutations at 2 specific residues in the Sel1 repeat of ExoR (G73 and S153) that are responsible for interaction with ExoS (ChvG) and result in increased ExoS(ChvG)/ChvI activity in *S. meliloti* [43]. We determined the effect on ChvG protein level and T6SS expression and secretion. ChvG-HA was co-expressed with wild-type ExoR, ExoR(G73C), ExoR(S153Y), or ExoR(G73C S153Y) in the *ΔchvG ΔexoR* mutant to determine whether these amino acid residues are critical for ChvG protein levels in neutral minimal medium. ChvG-HA protein level was lower with complementation of pExoR expressing wild-type ExoR than with the vector control (Figure 7A). ChvG-HA protein level was higher with overexpression of the variants ExoR(G73C), ExoR(S153Y), and ExoR(G73C S153Y) than with wild-type ExoR (Figure 7A). Furthermore, levels of proteins encoded by the *imp* and *hcp* operons were higher, and thus Hcp secretion, with the ExoR(G73C) and ExoR(S153Y) variants than with wild-type ExoR (Figure 7B and 7C). T6SS expression and secretion was no longer repressed with ExoR(G73C S153Y) expression (Figure 7B and 7C). Importantly, the abrogation of T6SS expression and secretion by ExoR was associated with its interaction with ChvG. As shown in our protein–protein interaction study with yeast two-hybrid assay (Figure 7D), we detected the interaction between the periplasmic domain of ChvG and wild-type ExoR but no or little interaction with the 3 ExoR variants that were compromised by its activity in negatively regulating T6SS.

The evidence that the amino acid residues of ExoR crucial for ExoR–ChvG interaction are also essential for ExoR in negatively regulating ChvG protein levels and abrogating downstream T6SS activation strongly argues that ExoR functions upstream of the ChvG/ChvI two-component system to negatively regulate T6SS by its association with ChvG sensor kinase in *A. tumefaciens*.

## Discussion

T6SS is a versatile protein secretion system highly regulated for bacterial survival or fitness. Many studies have uncovered the regulatory mechanisms of several T6SSs at multiple levels; however, the signals or molecular mechanisms involved in the regulation of most T6SSs remain largely unknown. Here, we discovered and provide the mechanistic details underlying how T6SS is regulated by an ExoR-ChvG/ChvI cascade in *A. tumefaciens* in response to pH change. In view of the acidic environment present in the wound site and apoplast of plants, a role of T6SS during *Agrobacterium*–plant interactions is strongly suggested but requires further study.

### T6SS is regulated by multiple environmental factors in *A. tumefaciens*

T6SS is involved in diverse functions, including promoting or repressing virulence, forming biofilm, and inducing cytotoxicity in eukaryotic or prokaryotic hosts. Many T6SSs of pathogenic bacteria are induced by host signals, which suggests their functions during bacteria–host interactions [6]. Several studies revealed that T6SS is tightly coordinated with other virulence determination systems such as T3SS, quorum sensing, and flagella synthesis [17], [57]–[59]. In *P. aeruginosa* and *Salmonella enterica*, T3SS and T6SS are inversely regulated to allow the transition from the acute to chronic virulence phase [24], [59], [60]. In *A. tumefaciens*, T6SS is regulated by multiple environmental factors, including pH and the virulence inducer we report. The abrogation of T6SS-mediated Hcp secretion by the addition of AS phenolics in an acidic minimal medium (AB-MES, pH 5.5) revealed an inverse association of T6SS and VirB/D4 T4SS that is highly induced by AS [31], [61]. Interestingly, Hcp secretion levels were greatly reduced in virulence-induced medium (AB-MES, pH 5.5, +AS) (Figure 1A), but the levels of all analyzed T6SS proteins except Hcp seemed to remain similar when grown with or without AS (Figure S1B), which suggests that AS might negatively regulate Hcp secretion posttranslationally. The slower migration of secreted Hcp protein as compared with cellular Hcp led us to explore the possibility of posttranslational modifications of Hcp such as phosphorylation. However, we detected no phosphorylation of Hcp (data not shown). The aberrant migration of secreted Hcp is likely caused by the presence of trichloroacetic acid (TCA) used for protein precipitated from culture medium because both cellular and secreted Hcp migrated slower in the presence of TCA (Figure S6).

Our previous study did not reveal the suppression of Hcp secretion at pH 7.0 [46]. The discrepancy is likely due to the use of a nutrient-rich 523 medium for overnight culture and the later time points for Hcp secretion assay in the previous work. Indeed, T6SS is almost silent when grown in neutral minimal medium (AB-MES, pH 7.0) (Figure 1), but its expression and secretion are active when grown in nutrient-rich medium such as 523 at pH 7.0 [46]. Thus, T6SS might be regulated by nutrient availability in *A. tumefaciens*. We also noted that T6SS secretion activity depends on growth phase, with Hcp secretion greatly reduced during the late logarithmic phase (J. Lin and E. Lai, unpublished results). Thus, T6SS is regulated by multiple factors via a complex regulatory network in a free-living environment or during *Agrobacterium*–plant interactions.

### T6SS is directly activated by the ChvG/ChvI two-component system by upregulating T6SS gene expression

By loss- and gain-of-function studies, we demonstrated that T6SS is regulated positively by the ChvG/ChvI two-component system and negatively by ExoR in *A. tumefaciens*. This discovery adds T6SS genes to the list of ChvG/ChvI-regulatory genes in *A. tumefaciens*. ExoR, ChvG/ChvI, and T6SS are widely distributed in *α-Proteobacteria*, which includes several animal and plant pathogens, as well as plant symbionts [32], [37], [45], [46], [62]–[64]. Thus, the regulation of T6SS via an ExoR-ChvG/ChvI cascade may be a universal regulatory mechanism in these bacteria.

The acid-induced T6SS gene expression and Hcp secretion is consistent with the acidity upregulation of *imp* genes found in previous microarray analyses [36], although the authors did not identify *hcp* operon-encoded genes as acidity-regulated genes. By investigating both the mRNA and protein levels in response to pH change together with loss- and gain-of-function studies, our data strongly argue that acid-induced *imp* operon expression is regulated at mRNA levels via transcriptional activation by the ChvG/ChvI two-component system. However, the *hcp* operon is regulated by a ChvG/ChvI pathway and by an unknown mechanism responsible for its basal expression even in the secretion-repression condition (AB-MES, pH 7.0). Thus, acid-induced T6SS secretion is mainly controlled by the expression of *imp*-encoding proteins constituting the T6S machinery. In addition, the higher induction of mRNA than protein levels by acidity (Figure 1) implicated additional regulation(s) of the *hcp* operon in translation efficiency and/or protein stability.

More strikingly, the phosphorylated state of ChvI was required for inducing *imp* operon expression and Hcp secretion but seemed to be dispensable for the increased expression of *hcp* operon (Figure 4). For the prototypical two-component system, the activated histidine sensor kinase phosphorylates the cognate response regulator at the conserved aspartate residue. The phosphorylation of the response regulator is generally required for binding to the target promoter [53]. The phosphorylation state of ChvI was indeed required for its binding to the intergenic region of the 2 operons *in vitro* and activated the expression of *imp* operon and T6SS secretion *in vivo*. Surprisingly, the overexpression of wild-type, phospho-mimic (D52E), or phospho-inactive (D52A) variants of ChvI could increase both mRNA and protein levels of *hcp* operon genes, which suggests that the phosphorylated state of ChvI is not required for upregulation of *hcp* operon expression. Because wild-type ChvI without detectable binding activity to the T6SS promoter region can enhance the level of proteins encoded by the *hcp* operon in neutral medium, where ChvG is repressed by ExoR, ChvI may not directly regulate the expression of the *hcp* operon. In view of the *chvG/chvI*-independent basal level expression of *hcp-*operon proteins, both phosphorylated and non-phosphorylated ChvI may positively influence the expression by interacting with a yet-to-be identified regulator(s) of *hcp* operon. Future investigation should aim to identify the cis-elements critical for ChvI binding and elucidate the molecular details of how ChvI coordinates the regulation of the expression of the 2 operons from the shared or overlapped regulatory region.

### Mechanisms of ExoR in derepressing ChvG for T6SS activation

Orthologs of ExoR and the ChvG/ChvI two-component system are present in many *α-Proteobacteria*, such as *Brucella*, *Bartonella*, *Sinorhizobium*, and *Rhizobium*, which suggests a conserved negative regulation of ChvG/ChvI by ExoR among these bacteria [65]. In *S. meliloti*, *exoR* is involved in sensing ammonia or calcium signals for derepressing the expression of ExoS/ChvI target genes *lpsS* and *exo* [66], [67]. Several two-component systems orthologous to ChvG/ChvI are activated in response to diverse signals; examples are *Bartonella henselae* BatR/BatS at the physiological pH of blood (pH 7.4) [40], *Brucella abortus* sensor kinase BvrS at the late logarithmic phase [68], and *A. tumefaciens* ChvG/ChvI in response to the acidic signal [36]. However, a clear ExoR–ChvG/ChvI regulatory cascade was demonstrated only in regulating the synthesis of exopolysaccharide succinoglycan, forming biofilm and motility in *S. meliloti* [41], [43] and regulating T6SS of *A. tumefaciens* in this study.

The epistasis of *chvG* to *exoR* in regulating T6SS expression and secretion indicates that *exoR* functions upstream of *chvG* in sensing acidity to regulate T6SS. The disruption of *A. tumefaciens* ExoR amino acid residues critical for interacting with ChvG causes the increased ChvG protein levels and T6SS expression/secretion, which suggests that ExoR negatively regulates ChvG/ChvI signaling by direct binding to ChvG. This conclusion is consistent with the finding that the physical interaction of ExoR–ExoS(ChvG) is important in repressing ExoS/ChvI activity in *S. meliloti* [43]. For unknown reasons, the ExoR protein levels differed between the wild type and variants (Figure 7A, 7C). However, the effect of ExoR variants on ChvG protein level and activating T6SS is indeed associated with the ability to interact with ChvG rather than the expression level (Figure 7). Together with the evidence that ChvG protein level is increased in the absence of *exoR*, ExoR may negatively regulate ChvG protein levels by directing binding.

The inverse association of ExoR protein level and ChvG protein stability in acidic conditions (overexpressed ExoR with unstable ChvG vs. endogenous ExoR with stable ChvG, Figure 5D, E) implied that ExoR negatively regulates ChvG protein stability. However, ChvG protein seems to retain similar stability independent of ExoR protein levels when grown in neutral medium. Moreover, ChvG protein is not more stable in acidic than neutral medium despite acidity inducing protein levels of ChvG when driven by a *lac* promoter (Figure 5B) or its own promoter on plasmid (Figure S7). Thus, the acid-induced ChvG protein accumulation cannot be simply explained by ExoR-regulated protein stability or its transcriptional activation (Figure 5) [36]. Our data suggest that additional regulations at posttranscriptional levels such as mRNA stability or translational control may also be involved but require future investigation.

Because endogenous ExoR is not detectable by antibody against *S. meliloti* ExoR or tagged with Strep epitope on chromosomes (Figure S7), ExoR proteins can be detected only by overexpression. However, the acid-triggered ExoRm degradation also likely occurs for endogenous ExoR in *A. tumefaciens* because of the following evidence. First, a proteomic study revealed reduced ExoR protein level after a pH shift from 7.0 to 5.5 in *Agrobacterium* sp. ATCC31750 [69]. Because *exoR* mRNA levels were not reduced in acidic medium (Figure 5A) [36], the levels of ExoR in acidic medium were not lowered by transcriptional regulation but were likely downregulated at the protein level. Furthermore, in *S. meliloti*, with detectable endogenous ExoR, a recent study revealed that periplasmic ExoRm is targeted for proteolysis and the ExoRm level regulates its role in suppressing ExoS (ChvG) activity [44]. Thus, if acid-triggered ExoRm degradation observed with overexpression indeed represents physiological relevance for endogenous ExoR, ExoR seems only to distablize ChvG in acidic medium, an adverse environment, where several proteases may be induced for protein quality control [70]. In this scenario, the increased protein levels of ectopically expressed ChvG in the absence of *exoR* or loss of binding to ExoR variants when grown in neutral medium must be regulated by mRNA stability and/or translational efficiency of *chvG*, but the potential regulations await future investigation.

**Figure 8:**
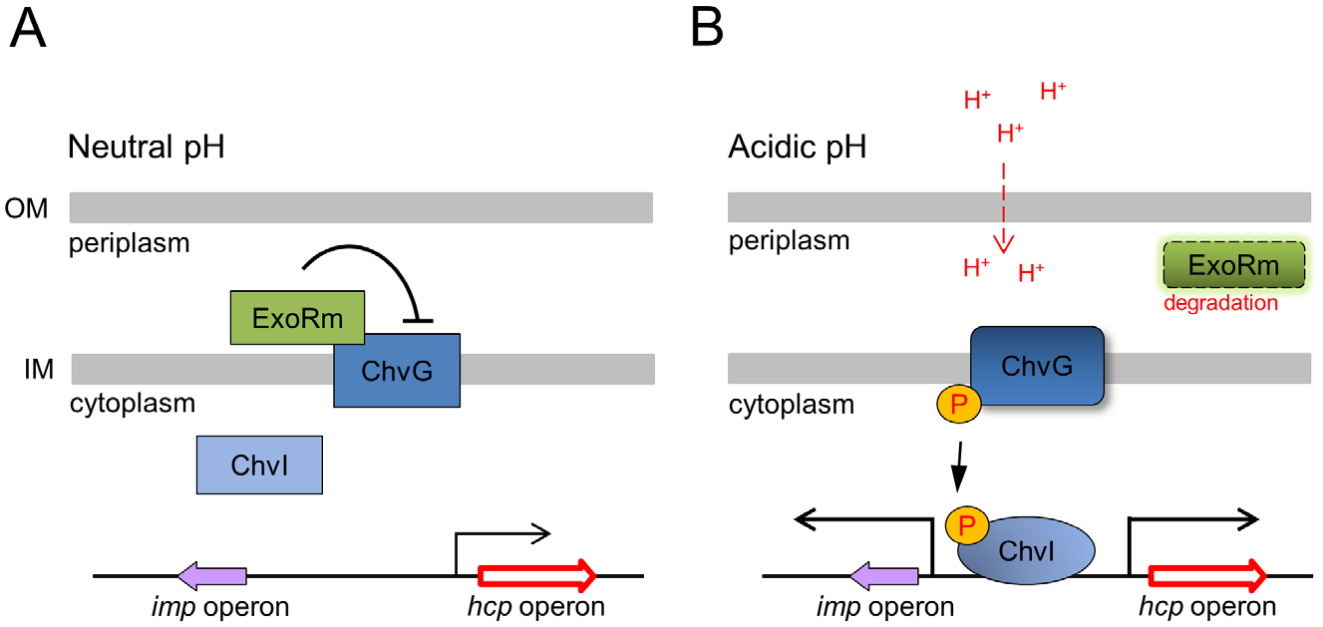
Model of ExoR-ChvG/ChvI-induced T6SS activation via acidity. (**A**) In neutral pH, ExoR associates with transmembrane sensor kinase ChvG and represses the ChvG/ChvI two-component system signaling, thus leading to reduced T6SS expression and secretion activity. (**B**) In acidic pH, ExoR loses its activity in repressing ChvG sensor kinase, which is then activated to phosphorylate the response regulator ChvI. The phosphorylated ChvI positively regulates T6SS expression, thus resulting in activation of T6SS secretion. Acidic signal may trigger the degradation or possible conformational change of ExoRm and thereby derepress ChvG for T6SS activation. The size and width of arrow above the *imp* and *hcp* operons represent the level of expression. OM, outer membrane; IM, inner membrane.

To this end, we propose that acid-induced ExoRm degradation is responsible or one factor for the increased ChvG protein levels or activity and T6SS activation. As illustrated in our proposed model (Figure 8), in the absence of an acidic signal, periplasmic ExoRm interacts with the ChvG sensor kinase located in the inner membrane, thus leading to inhibited ChvG/ChvI signaling pathways, including T6SS. When *A. tumefaciens* senses the acidic signal, ExoR loses its activity in repressing ChvG, thereby allowing the ChvG sensor kinase to relay ChvI signaling for T6SS activation. Periplasmic ExoRm may be more stable in neutral pH and rapidly degraded when sensing acidity. However, whether acid-triggered ExoRm degradation is the key or responsible for activating ChvG/ChvI signaling is not yet verified. We do not exclude that acid may also induce a conformational change of ExoR or even ChvG to regulate ChvG/ChvI signaling. Thus, it remains important to visualize the endogenous expression of ExoR and ChvG in response pH changes by pulse-chase analysis [71] or quantitation by mass spectrometry [72]. Identifying the protease responsible for ExoRm degradation and/or proteolysis-resistant variants for ExoR and ChvG will be the key for a firm conclusion for the proposed mechanism. Whether acid-induced T6SS is a common regulatory mechanism in plant-associated bacteria and the biological significance of this regulation are important questions for future investigation.

## Material and Methods

### Plasmids, bacterial strains, and growth conditions

Plasmids, bacterial strains and primers are in supplemental Tables S1 and S2. *A. tumefaciens* strains were grown in 523 medium [73]; *ΔchvG*, *ΔchvI*, and *ΔchvG ΔexoR* mutant strains were grown in AB-MES minimal medium, which contains 3 g K_2_HPO_4_, 1 g NaH_2_PO_4_, 1 g NH_4_Cl, 0.15 g KCl, 0.01 g CaCl2, 0.3 g MgSO_4_-7H_2_O, 2.5 mg FeSO_4_-7H_2_O, 9.76 g 2-(*N*-morpholine)ethanesulfonic acid, and 20 g glucose per liter (pH 7.0) [74]. For T6SS expression and secretion analysis, *A. tumefaciens* cells grown overnight in AB-MES medium (pH 7.0) with appropriate antibiotics were harvested by centrifugation (8,000× g, 10 min) and resuspended in AB-MES medium (pH 7.0 or 5.5) without any antibiotics at ∼0.1 optical density 600 nm (OD_600_). After growth for 6 h at 25°C, cells were harvested, and proteins secreted into the culture medium were precipitated with trichloroacetic acid (TCA) as described [46], [47]. For western blot analysis of ChvG-HA and ExoR, cells were harvested after growth for 3, 6, 12, and 24 h at 25°C. For ExoR protein stability analysis, *A. tumefaciens* cells were resuspended in AB-MES medium (pH 7.0 or 5.5) containing chloramphenicol (100 µg/ml) at OD_600_ ∼0.5 from *A. tumefaciens* cells grown overnight in AB-MES medium (pH 7.0) with appropriate antibiotics. Cells were harvested after growth for 1, 2, and 3 h at 25°C. For *A. tumefaciens*, the concentration of the antibiotic gentamycin was 50 µg/ml; spectinomycin, 250 µg/ml; and chloramphenicol, 100 µg/ml, and for *Escherichia coli*, the concentration for gentamycin was 50 µg/ml; spectinomycin, 100 µg/ml; and kanamycin, 20 µg/ml.

### Plasmid construction

The plasmids pJQ200KS-Δ*chvG*, pJQ200KS-Δ*chvI*, and pJQ200KS-Δ*exoR* were created by ligating the *Xba*I/*Bam*HI-digested PCR product 1 (∼500 bp DNA fragments upstream of each target gene) and *Bam*HI/*Xma*I-digested PCR product 2 (∼500 bp DNA fragments downstream of each target gene) into *Xba*I/*Xma*I sites of pJQ200KS [75] to generate each of the deletion mutants, for which at least 2 independent colonies were selected and confirmed by PCR.

The PCR products of *chvG* and *chvI* genes containing the ribosomal-binding sequence (RBS) and open reading frame (ORF) were digested by *Xho*I/*Xba*I and cloned into the same sites of pRL662 [76], which resulted in the plasmids pChvG and pChvI. To construct the plasmid for ChvG tagged with HA, the *chvG* DNA fragment containing the RBS and ORF (without a stop codon) was PCR-amplified, digested by *Sac*I/*Xba*I, and cloned into the same sites of pBluescript SK(+)-HA. The resulting plasmids were further digested by *Xho*I/*Hin*dIII and cloned into the same sites of pRL662 to create the plasmid pChvG-HA.

The PCR products of *exoR* containing its RBS and ORF were digested by *Xma*I/*Xba*I and cloned into the same sites of pTrc200 [77], which resulted in the plasmid pExoR. The expression vector pET22b(+) (Novagen) was used to overexpress proteins driven by the T7 promoter via isopropyl-beta-D-thiogalactoside (IPTG) induction in *E. coli* BL21(DE3) [78]. Each ORF (without a stop codon) of *fha1*, *atu4343*, *atu4349*, and *rpoA* was PCR-amplified and cloned into the same sites of pET22b(+) with appropriate enzyme sites. The *clpV* ORF (without a stop codon) was PCR-amplified, digested by *Hin*dIII, and cloned into pET22b(+), which was first digested by *Nde*I, followed by Klenow repair, and finally digested by *Hin*dIII. N-terminal His-tagged wild-type and D52E ChvI were constructed by PCR amplification of the full-length wild-type or D52E ChvI with flanking *Nde*I/*Xho*I restriction sites and cloning into pET28a (Novagen).

For the constructs used for yeast two-hybrid, various *exoR* ORFs were PCR-amplified (by primers AD ExoR F & AD ExoR R), digested (*Nde*I/*Xho*I), and cloned into the *Nde*I/*Xho*I sites of pGADT7 for N-terminal fusion to the activation domain (AD), pGADT7-ExoR, pGADT7-ExoR^G73C^, pGADT7-ExoR^S153Y^ and pGADT7-ExoR^G73C/S153Y^. For plasmid pGBKT7-ChvG-peri, the DNA fragment encoding the periplasmic domain of ChvG (71–278 a.a.) was PCR-amplified with primers BD ChvGperi F & BD ChvGperi R, digested with *Nde*I/*Bam*HI, and cloned into the same sites of pGBKT7.

### Quantitative real-time PCR (qRT-PCR)

Total RNA from *A. tumefaciens* strains grown in AB-MES minimal medium (pH 7.0 or pH 5.5) was extracted by the hot-phenol method [79] and treated with DNase I (Promega) to eliminate DNA contamination. RNA was reverse transcribed with random oligonucleotide hexamers (Promega) and the SuperScript III Reverse Transcriptase method (Invitrogen). qRT-PCR involved use of specific primers with Power SYBR Green PCR Master Mix reagent (Applied Biosystems) and the ABI 7500 Real-Time PCR System (Applied Biosystems). The program for qRT-PCR was 2 min at 50°C, 10 min at 95°C, 40 cycles of 15 s at 95°C/1 min at 60°C. Expression was normalized to that of 16S rRNA as an internal control by the 2^−*ΔΔ*Ct^ method [80].

### Generation of in-frame deletion mutants

All in-frame deletion mutants were generated in *A. tumefaciens* C58 via double crossover with the suicide plasmid pJQ200KS [75] as described [46], [47].

### Antibody production

The detailed methods of plasmid construction, overexpression, and purification of His-tagged Fha1, Atu4343, ClpV, Atu4349, and RpoA proteins for antibody production will be published elsewhere (J. Lin and E. Lai, unpublished results). In brief, the expression vector pET22b(+) was used to overexpress His-tagged proteins driven by the T7 promoter with IPTG induction in *E. coli* BL21(DE3) followed by purification with an Ni^2+^-NTA column (Novagen) as described [46], [47]. Purified proteins were separated by glycine-SDS-PAGE, and the protein band was cut out to obtain polyclonal antibody in rabbits.

### Overexpression and purification of His-tagged ChvI protein in *E. coli*

The pET28a-ChvI and pET28a-ChvI(D52E) plasmids were transformed into *E. coli* BL21(DE3) cells for protein expression. Briefly, cultures were induced with 0.4 mM IPTG for 3 h at 37°C, and cells were lysed by use of the French Press (Aminco, Silver Spring, MD, USA) as described [46], [47].

### Western blot analysis

Proteins were resolved by 13% glycine-SDS-PAGE and western blot analysis was performed as described [81] with primary polyclonal antibodies produced in this study and against C-IcmF [47], Hcp [46], ActC [82], VirE2 [83] encoded by *A. tumefaciens*; ExoR [44] encoded by *S. meliloti;* or monoclonal antibody against HA (Sigma). The secondary antibody was horseradish peroxidase-conjugated goat anti-rabbit or anti-rabbit IgG (Chemicon), and signals were detected by use of the Western Lightning System (Perkin Elmer, Boston, MA). Chemiluminescent bands were visualized by use of X-ray film (Kodak, Rochester, NY) or the BioSpectrum AC Imaging System (Ultra-Violet Products Ltd., UK) to detect and quantify the photon intensity of protein signals.

### Electrophoretic mobility shift assay (EMSA)

The 230-bp intergenic region of the *imp* and *hcp* operons and the control DNA fragment were PCR-amplified with the primers T6SSF/T6SSR and Atu4353F/Atu4353R, respectively, and purified by use of the PCR DNA fragment extraction kit (Geneaid, Taiwan). The T6SS regulatory region and control DNA fragments were digested with *Xho*I and *Bam*HI, respectively, and filled in with [*α*-^32^P]dCTP and unlabeled dTTP, dATG, and dGTG with the klenow fragment of DNA polymerase I. The labeled ^32^P-labeled DNA fragments were purified by use of G50 Mini columns (Geneaid). Labeled DNA fragments (1.5 ng) were incubated with purified ChvI protein (11 to 150 ng) in 10 µl binding buffer (10 mM Tris-Cl [pH 7.5], 1 mM EDTA, 0.1 mM dithiothreitol, 5% glycerol, 0.05 mg/ml bovine serum albumin [BSA]) for 20 min at room temperature and then analyzed on 5% Tris-borate-EDTA non-denaturing acrylamide gels at 4°C. The separated DNA–protein complex was dried by use of a gel dryer and visualized by use of X-ray film (Kodak).

### Site-directed mutagenesis

The DNA fragment encoding ChvI(D52E), ChvI(D52A), ExoR(G73C), or ExoR(S153Y) mutations was created by PCR-based site-directed mutagenesis as described [84]. The *chvI*(*D52E*) and *chvI*(*D52A*) DNA fragments were digested by *Nde*I/*Xba*I and cloned into the same sites of pRL662 to create the plasmids pChvI(D52E) and pChvI(D52A). The *exoR*(*G73C*), *exoR*(*S153Y*), and *exoR*(*G73C S153Y*) DNA fragments were digested by *Xma*I/*Xba*I and cloned into the same sites of pRL662 to create the plasmids pExoR(G73C), pExoR(S153Y), and pExoR(G73C S153Y).

### Biochemical fractionation

Isolation of *A. tumefaciens* cellular fractions was as described [47].

### Yeast two-hybrid assay

The Matchmaker yeast two-hybrid system was used according to the user manual instructions (Clontech, Mountain View, CA). Each of the plasmid pairs were co-transformed into *Saccharomyces cerevisiae* strain AH109. The transformants were selected by their growth on synthetic dextrose (SD) minimal medium lacking tryptophan (Trp) and leucine (Leu) (SD-WL medium). The positive interaction of expressed fusion proteins was determined by their growth on SD lacking Trp, Leu, adenine (Ade), and histidine (His) (SD-WLHA medium) at 30°C for at least 2 days.

### Accession numbers of genes and proteins

Genebank accession numbers for genes: *hcp* (1136219), *rpoA* (1133961), *virE2* (1137513), *icmF* (1136206), *fha1* (1136209), *atu4343* (1136217), *clpV* (1136218), *atu4349* (1136223), *chvG* (1132071), *chvI* (1132072), *exoR* (1133753), *chvH* (1134591)

Genebank accession numbers for proteins: Hcp (NP_356310), RpoA (NP_354899), VirE2 (NP_396510), IcmF (NP_356323), Fha1 (NP_356320), Atu4343 (NP_356312), ClpV (NP_356311), Atu4349 (NP_356306), ChvG (NP_353072), ChvI (NP_353073), ExoR (NP_354703), ChvH (NP_355493)

## Supporting Information

Figure S1 **The effects of acetosyringone (AS) and different carbon sources on T6SS expression and Hcp secretion.** (**A**) Total proteins isolated from *Agrobacterium tumefaciens* wild-type C58 grown in AB-MES (pH 7.0 or pH 5.5) supplemented with indicated carbon sources at 25°C for 6 h were resolved by glycine-SDS-PAGE, followed by western blot analysis with antibodies against C-IcmF, Fha1 (filled arrow), Atu4343, ClpV, Hcp, Atu4349, and RpoA. (**B**) Total and secreted (Sup) proteins isolated from C58 grown in AB-MES (pH 7.0 or pH 5.5) with glycerol, DMSO (used to dissolve acetosyringone [AS]), or 200 µM AS at 25°C for 6 h were resolved by glycine-SDS-PAGE, followed by western blot analysis with antibodies against C-IcmF, Fha1 (filled arrow), Atu4343, ClpV, Hcp, Atu4349, VirE2, and RpoA. RpoA was used as an internal control. The positions of molecular mass markers (in kDa) are indicated on the left.
(TIF)

Figure S2 **T6SS expression and Hcp secretion are concomitantly upregulated by acidity in** ***A. tumefaciens*** **Ach5 and 1D1609 strains.** (**A**) Secreted (Sup) proteins isolated from *A. tumefaciens* wild-type C58, Ach5, 1D1609 strains grown in AB-MES (pH 7.0 or pH 5.5) at 25°C for 6 h were resolved by glycine-SDS-PAGE, followed by western blot analysis with antibodies against Hcp and RpoA. (**B**) Total proteins isolated from the strains and conditions described in (**A**) were resolved by glycine-SDS-PAGE, followed by western blot analysis with antibodies against C-IcmF, Fha1 (filled arrow), Atu4343, ClpV, Hcp, and RpoA. RpoA is a non-secreted protein used as an internal control. The positions of molecular mass markers (in kDa) are indicated on the left.
(TIF)

Figure S3 **EMSA of the binding specificity of ChvI and T6SS regulatory region.** The ^32^P-labeled 230-bp intergenic region between the *imp* and *hcp* operons or DNA fragment derived from the *atu4353* coding region was incubated without or with increasing levels of purified recombinant variant ChvI(D52E) protein (50 and 150 ng). The shifted band is indicated by *.
(TIF)

Figure S4 **Quantification of levels of** ***hcp*** **operon-encoded proteins with overexpression of ChvI variants.** Total proteins isolated from *A. tumefaciens* C58 strains containing the empty vector (V) or one of the plasmids expressing ChvI, ChvI(D52E) or ChvI(D52A) grown in AB-MES (pH 7.0) at 25°C for 6 h underwent western blot analysis with antibodies against ClpV, Hcp, and Atu4349. Protein levels of ClpV, Hcp, and Atu4349 were quantified with use of the UVP BioSpectrum 600 Imaging System and normalized to the level of endogenous RpoA. The level of vector control was set to 1. Data are mean ± SD of two biological replicates.
(TIF)

Figure S5 **Alignment of the amino acid sequences of** ***A. tumefaciens*** **and** ***S. meliloti*** **ExoR.** ExoR orthologs from Atume (*A. tumefaciens;* Genbank accession no. NP_354703) and Smeli (*S. meliloti*; AAA26260) were aligned with 74% identity. The identical amino acid residues are highlighted in black, and the arrow indicates a predicted signal peptide cleavage site. The amino acid residues used for mutagenesis are indicated.
(TIF)

Figure S6 **Effect of trichloroacetic acid on migration of cellular and secreted Hcp proteins.** (**A**) Total (T) and secreted (S) proteins were isolated from *A. tumefaciens* wild-type C58 grown in AB-MES (pH 5.5) at 25°C for 6 h. The supernatant was precipitated by TCA without washing or followed by 85% acetone wash. The proteins were analyzed by western blot analysis with antibodies against Hcp and ActC. The periplasmic protein ActC was an internal control. (**B**) Western blot analysis of total protein levels of Hcp or RpoA with or without 10% TCA.
(TIF)

Figure S7 **Expression analysis of ChvG and ExoR driven by native promoters.** (**A**) *A. tumefaciens ΔchvG* expressing pChvI-ChvG-HA driven by P*chvIG* native promoter on plasmid pRL662 was grown in AB-MES (pH 7.0 or pH 5.5) at 25°C for the indicated times. Total proteins were analyzed by western blot analysis with antibodies against HA (for detecting ChvG-HA) and RpoA. The positions of molecular mass markers (in kDa) are indicated on the left. (**B**) PCR confirmation of *exoR-strep* expressed from chromosomal locus. The wild-type *exoR* gene was replaced by the Strep-tagged *exoR* with confirmation by PCR analysis with primers corresponding to the *exoR* coding region and the downstream flanking region. A 130-bp product was identified in wild-type C58 (lane 1) and a 154-bp product in the strain with *exoR-strep* gene replacement (lane 2–5). (**C**) Western blot analysis of protein level of ExoR-Strep. Total proteins isolated from *A. tumefaciens* wild-type C58 strain and 4 colonies of the strain grown in AB-MES (pH 7.0) overnight at 25°C underwent western blotting with antibodies against strep (for detecting ExoR-strep) and RpoA. ExoR-Strep was detected from expression with the plasmid form (lane 6 *ΔexoR* containing pTrC-ExoR-Strep) but not from wild type C58 (lane 1) or the 4 independent colonies with chromosomal fusion (lanes 2, 3, 4, 5).
(TIF)

Table S1 **Bacterial strains and plasmids.**
(PDF)

Table S2 **Primers used in this study.**
(PDF)

## References

[1] Holland IB (2010) The extraordinary diversity of bacterial protein secretion mechanisms. Methods Mol Biol 619: 1–20. 20419401 10.1007/978-1-60327-412-8_1

[2] Boyer F, Fichant G, Berthod J, Vandenbrouck Y, Attree I (2009) Dissecting the bacterial type VI secretion system by a genome wide in silico analysis: what can be learned from available microbial genomic resources? BMC Genomics 10: 104. 19284603 10.1186/1471-2164-10-104 PMC2660368

[3] Cascales E (2008) The type VI secretion toolkit. EMBO Rep 9: 735–741. 18617888 10.1038/embor.2008.131 PMC2515208

[4] Filloux A, Hachani A, Bleves S (2008) The bacterial type VI secretion machine: yet another player for protein transport across membranes. Microbiology 154: 1570–1583. 18524912 10.1099/mic.0.2008/016840-0

[5] Records AR (2011) The type VI secretion system: a multipurpose delivery system with a phage-like machinery. Mol Plant Microbe Interact 24: 751–757. 21361789 10.1094/MPMI-11-10-0262

[6] Schwarz S, Hood RD, Mougous JD (2010) What is type VI secretion doing in all those bugs? Trends Microbiol 18: 531–537. 20961764 10.1016/j.tim.2010.09.001 PMC2991376

[7] Chong A, Wehrly TD, Nair V, Fischer ER, Barker JR, et al (2008) The early phagosomal stage of Francisella tularensis determines optimal phagosomal escape and Francisella pathogenicity island protein expression. Infect Immun 76: 5488–5499. 18852245 10.1128/IAI.00682-08 PMC2583578

[8] Pukatzki S, Ma AT, Sturtevant D, Krastins B, Sarracino D, et al (2006) Identification of a conserved bacterial protein secretion system in Vibrio cholerae using the Dictyostelium host model system. Proc Natl Acad Sci U S A 103: 1528–1533. 16432199 10.1073/pnas.0510322103 PMC1345711

[9] Schwarz S, West TE, Boyer F, Chiang WC, Carl MA, et al (2010) Burkholderia type VI secretion systems have distinct roles in eukaryotic and bacterial cell interactions. PLoS Pathog 6: e1001068. 20865170 10.1371/journal.ppat.1001068 PMC2928800

[10] Russell AB, Hood RD, Bui NK, LeRoux M, Vollmer W, et al (2011) Type VI secretion delivers bacteriolytic effectors to target cells. Nature 475: 343–347. 21776080 10.1038/nature10244 PMC3146020

[11] Weber B, Hasic M, Chen C, Wai SN, Milton DL (2009) Type VI secretion modulates quorum sensing and stress response in Vibrio anguillarum. Environ Microbiol 11: 3018–3028. 19624706 10.1111/j.1462-2920.2009.02005.x

[12] de Pace F, Boldrin de Paiva J, Nakazato G, Lancellotti M, Sircili MP, et al (2011) Characterization of IcmF of the type VI secretion system in an avian pathogenic Escherichia coli (APEC) strain. Microbiology 157: 2954–2962. 21778203 10.1099/mic.0.050005-0 PMC3353391

[13] Burtnick MN, DeShazer D, Nair V, Gherardini FC, Brett PJ (2010) Burkholderia mallei cluster 1 type VI secretion mutants exhibit growth and actin polymerization defects in RAW 264.7 murine macrophages. Infect Immun 78: 88–99. 19884331 10.1128/IAI.00985-09 PMC2798217

[14] Basler M, Pilhofer M, Henderson GP, Jensen GJ, Mekalanos JJ (2012) Type VI secretion requires a dynamic contractile phage tail-like structure. Nature 483: 182–186. 22367545 10.1038/nature10846 PMC3527127

[15] Brunet YR, Bernard CS, Gavioli M, Lloubes R, Cascales E (2011) An epigenetic switch involving overlapping fur and DNA methylation optimizes expression of a type VI secretion gene cluster. PLoS Genet 7: e1002205. 21829382 10.1371/journal.pgen.1002205 PMC3145626

[16] Bernard CS, Brunet YR, Gueguen E, Cascales E (2010) Nooks and crannies in type VI secretion regulation. J Bacteriol 192: 3850–3860. 20511495 10.1128/JB.00370-10 PMC2916374

[17] Leung KY, Siame BA, Snowball H, Mok YK (2011) Type VI secretion regulation: crosstalk and intracellular communication. Curr Opin Microbiol 14: 9–15. 20971679 10.1016/j.mib.2010.09.017

[18] Castang S, McManus HR, Turner KH, Dove SL (2008) H-NS family members function coordinately in an opportunistic pathogen. Proc Natl Acad Sci U S A 105: 18947–18952. 19028873 10.1073/pnas.0808215105 PMC2596223

[19] Ishikawa T, Rompikuntal PK, Lindmark B, Milton DL, Wai SN (2009) Quorum sensing regulation of the two hcp alleles in Vibrio cholerae O1 strains. PLoS One 4: e6734. 19701456 10.1371/journal.pone.0006734 PMC2726435

[20] Schell MA, Ulrich RL, Ribot WJ, Brueggemann EE, Hines HB, et al (2007) Type VI secretion is a major virulence determinant in Burkholderia mallei. Mol Microbiol 64: 1466–1485. 17555434 10.1111/j.1365-2958.2007.05734.x

[21] Dudley EG, Thomson NR, Parkhill J, Morin NP, Nataro JP (2006) Proteomic and microarray characterization of the AggR regulon identifies a pheU pathogenicity island in enteroaggregative Escherichia coli. Mol Microbiol 61: 1267–1282. 16925558 10.1111/j.1365-2958.2006.05281.x

[22] Bernard CS, Brunet YR, Gavioli M, Lloubes R, Cascales E (2011) Regulation of type VI secretion gene clusters by sigma54 and cognate enhancer binding proteins. J Bacteriol 193: 2158–2167. 21378190 10.1128/JB.00029-11 PMC3133059

[23] Wang X, Wang Q, Xiao J, Liu Q, Wu H, et al (2009) Edwardsiella tarda T6SS component evpP is regulated by esrB and iron, and plays essential roles in the invasion of fish. Fish Shellfish Immunol 27: 469–477. 19563898 10.1016/j.fsi.2009.06.013

[24] Parsons DA, Heffron F (2005) sciS, an icmF homolog in Salmonella enterica serovar Typhimurium, limits intracellular replication and decreases virulence. Infect Immun 73: 4338–4345. 15972528 10.1128/IAI.73.7.4338-4345.2005 PMC1168621

[25] Brencic A, Lory S (2009) Determination of the regulon and identification of novel mRNA targets of Pseudomonas aeruginosa RsmA. Mol Microbiol 72: 612–632. 19426209 10.1111/j.1365-2958.2009.06670.x PMC5567987

[26] Mougous JD, Gifford CA, Ramsdell TL, Mekalanos JJ (2007) Threonine phosphorylation post-translationally regulates protein secretion in Pseudomonas aeruginosa. Nat Cell Biol 9: 797–803. 17558395 10.1038/ncb1605

[27] Citovsky V, Kozlovsky SV, Lacroix B, Zaltsman A, Dafny-Yelin M, et al (2007) Biological systems of the host cell involved in Agrobacterium infection. Cell Microbiol 9: 9–20. 17222189 10.1111/j.1462-5822.2006.00830.x

[28] McCullen CA, Binns AN (2006) Agrobacterium tumefaciens and plant cell interactions and activities required for interkingdom macromolecular transfer. Annu Rev Cell Dev Biol 22: 101–127. 16709150 10.1146/annurev.cellbio.22.011105.102022

[29] Gelvin SB (2010) Plant proteins involved in Agrobacterium-mediated genetic transformation. Annu Rev Phytopathol 48: 45–68. 20337518 10.1146/annurev-phyto-080508-081852

[30] Doty SL, Yu MC, Lundin JI, Heath JD, Nester EW (1996) Mutational analysis of the input domain of the VirA protein of Agrobacterium tumefaciens. J Bacteriol 178: 961–970. 8576069 10.1128/jb.178.4.961-970.1996 PMC177754

[31] Alvarez-Martinez CE, Christie PJ (2009) Biological diversity of prokaryotic type IV secretion systems. Microbiol Mol Biol Rev 73: 775–808. 19946141 10.1128/MMBR.00023-09 PMC2786583

[32] Li L, Jia Y, Hou Q, Charles TC, Nester EW, et al (2002) A global pH sensor: Agrobacterium sensor protein ChvG regulates acid-inducible genes on its two chromosomes and Ti plasmid. Proc Natl Acad Sci U S A 99: 12369–12374. 12218184 10.1073/pnas.192439499 PMC129451

[33] Mantis NJ, Winans SC (1993) The chromosomal response regulatory gene chvI of Agrobacterium tumefaciens complements an Escherichia coli phoB mutation and is required for virulence. J Bacteriol 175: 6626–6636. 8407840 10.1128/jb.175.20.6626-6636.1993 PMC206774

[34] Liu P, Wood D, Nester EW (2005) Phosphoenolpyruvate carboxykinase is an acid-induced, chromosomally encoded virulence factor in Agrobacterium tumefaciens. J Bacteriol 187: 6039–6045. 16109945 10.1128/JB.187.17.6039-6045.2005 PMC1196135

[35] Jia YH, Li LP, Hou QM, Pan SQ (2002) An Agrobacterium gene involved in tumorigenesis encodes an outer membrane protein exposed on the bacterial cell surface. Gene 284: 113–124. 11891052 10.1016/s0378-1119(02)00385-2

[36] Yuan ZC, Liu P, Saenkham P, Kerr K, Nester EW (2008) Transcriptome profiling and functional analysis of Agrobacterium tumefaciens reveals a general conserved response to acidic conditions (pH 5.5) and a complex acid-mediated signaling involved in Agrobacterium-plant interactions. J Bacteriol 190: 494–507. 17993523 10.1128/JB.01387-07 PMC2223696

[37] Charles TC, Nester EW (1993) A Chromosomally Encoded 2-Component Sensory Transduction System Is Required for Virulence of Agrobacterium-Tumefaciens. J Bacteriol 175: 6614–6625. 8407839 10.1128/jb.175.20.6614-6625.1993 PMC206773

[38] Cheng HP, Walker GC (1998) Succinoglycan production by Rhizobium meliloti is regulated through the ExoS-ChvI two-component regulatory system. J Bacteriol 180: 20–26. 9422587 10.1128/jb.180.1.20-26.1998 PMC106843

[39] Sola-Landa A, Pizarro-Cerda J, Grillo MJ, Moreno E, Moriyon I, et al (1998) A two-component regulatory system playing a critical role in plant pathogens and endosymbionts is present in Brucella abortus and controls cell invasion and virulence. Mol Microbiol 29: 125–138. 9701808 10.1046/j.1365-2958.1998.00913.x

[40] Quebatte M, Dehio M, Tropel D, Basler A, Toller I, et al (2010) The BatR/BatS two-component regulatory system controls the adaptive response of Bartonella henselae during human endothelial cell infection. J Bacteriol 192: 3352–3367. 20418395 10.1128/JB.01676-09 PMC2897681

[41] Wells DH, Chen EJ, Fisher RF, Long SR (2007) ExoR is genetically coupled to the ExoS-ChvI two-component system and located in the periplasm of Sinorhizobium meliloti. Mol Microbiol 64: 647–664. 17462014 10.1111/j.1365-2958.2007.05680.x

[42] Lu HY, Cheng HP (2010) Autoregulation of Sinorhizobium meliloti exoR gene expression. Microbiology 156: 2092–2101. 20413557 10.1099/mic.0.038547-0 PMC3068678

[43] Chen EJ, Sabio EA, Long SR (2008) The periplasmic regulator ExoR inhibits ExoS/ChvI two-component signalling in Sinorhizobium meliloti. Mol Microbiol 69: 1290–1303. 18631237 10.1111/j.1365-2958.2008.06362.x PMC2652646

[44] Lu HY, Luo L, Yang M, Cheng HP (2012) Sinorhizobium meliloti ExoR is the target of periplasmic proteolysis. J Bacteriol 194: 4029–40. 22636773 10.1128/JB.00313-12 PMC3416547

[45] Tomlinson AD, Ramey-Hartung B, Day TW, Merritt PM, Fuqua C (2010) Agrobacterium tumefaciens ExoR represses succinoglycan biosynthesis and is required for biofilm formation and motility. Microbiology 156: 2670–2681. 20576688 10.1099/mic.0.039032-0 PMC3068688

[46] Wu HY, Chung PC, Shih HW, Wen SR, Lai EM (2008) Secretome analysis uncovers an Hcp-family protein secreted via a type VI secretion system in Agrobacterium tumefaciens. J Bacteriol 190: 2841–2850. 18263727 10.1128/JB.01775-07 PMC2293243

[47] Ma LS, Lin JS, Lai EM (2009) An IcmF family protein, ImpLM, is an integral inner membrane protein interacting with ImpKL, and its walker a motif is required for type VI secretion system-mediated Hcp secretion in Agrobacterium tumefaciens. J Bacteriol 191: 4316–4329. 19395482 10.1128/JB.00029-09 PMC2698499

[48] Ma LS, Narberhaus F, Lai EM (2012) IcmF family protein TssM exhibits ATPase activity and energizes type VI secretion. J Biol Chem 287: 15610–15621. 22393043 10.1074/jbc.M111.301630 PMC3346141

[49] Zheng J, Leung KY (2007) Dissection of a type VI secretion system in Edwardsiella tarda. Mol Microbiol 66: 1192–1206. 17986187 10.1111/j.1365-2958.2007.05993.x

[50] Zheng J, Ho B, Mekalanos JJ (2011) Genetic Analysis of Anti-Amoebae and Anti-Bacterial Activities of the Type VI Secretion System in Vibrio cholerae. PLoS One 6: e23876. 21909372 10.1371/journal.pone.0023876 PMC3166118

[51] Palumbo JD, Phillips DA, Kado CI (1998) Characterization of a new Agrobacterium tumefaciens strain from alfalfa. Arch Microbiol 169: 381–386. 9560417 10.1007/s002030050586

[52] Lin BC, Kado CI (1977) Studies on *Agrobacterium tumefaciens*. VIII. Avirulence induced by temperature and ethidium bromide. Can J Microbiol 23: 1554–1561. 922605 10.1139/m77-229

[53] Mitrophanov AY, Groisman EA (2008) Signal integration in bacterial two-component regulatory systems. Genes Dev 22: 2601–2611. 18832064 10.1101/gad.1700308 PMC2751022

[54] Klose KE, Weiss DS, Kustu S (1993) Glutamate at the site of phosphorylation of nitrogen-regulatory protein NTRC mimics aspartyl-phosphate and activates the protein. J Mol Biol 232: 67–78. 8331671 10.1006/jmbi.1993.1370

[55] Chen EJ, Fisher RF, Perovich VM, Sabio EA, Long SR (2009) Identification of direct transcriptional target genes of ExoS/ChvI two-component signaling in Sinorhizobium meliloti. J Bacteriol 191: 6833–6842. 19749054 10.1128/JB.00734-09 PMC2772461

[56] Mittl PR, Schneider-Brachert W (2007) Sel1-like repeat proteins in signal transduction. Cell Signal 19: 20–31. 16870393 10.1016/j.cellsig.2006.05.034

[57] Zheng J, Tung SL, Leung KY (2005) Regulation of a type III and a putative secretion system in Edwardsiella tarda by EsrC is under the control of a two-component system, EsrA-EsrB. Infect Immun 73: 4127–4137. 15972502 10.1128/IAI.73.7.4127-4137.2005 PMC1168592

[58] Syed KA, Beyhan S, Correa N, Queen J, Liu J, et al (2009) The Vibrio cholerae flagellar regulatory hierarchy controls expression of virulence factors. J Bacteriol 191: 6555–6570. 19717600 10.1128/JB.00949-09 PMC2795290

[59] Moscoso JA, Mikkelsen H, Heeb S, Williams P, Filloux A (2011) The Pseudomonas aeruginosa sensor RetS switches type III and type VI secretion via c-di-GMP signalling. Environ Microbiol 13: 3128–3138. 21955777 10.1111/j.1462-2920.2011.02595.x

[60] Mougous JD, Cuff ME, Raunser S, Shen A, Zhou M, et al (2006) A virulence locus of Pseudomonas aeruginosa encodes a protein secretion apparatus. Science 312: 1526–1530. 16763151 10.1126/science.1128393 PMC2800167

[61] Stachel SE, Messens E, Montagu MV, Zambryski P (1985) identification of the signal molecules produced by wounded plant cells that activate T-DNA transfer in Agrobacterium tumefaciens. Nature 318: 624–629.

[62] Reeve WG, Dilworth MJ, Tiwari RP, Glenn AR (1997) Regulation of exopolysaccharide production in Rhizobium leguminosarum biovar viciae WSM710 involves exoR. Microbiology 143 Pt 6: 1951–1958. 9202471 10.1099/00221287-143-6-1951

[63] Vanderlinde EM, Yost CK (2012) Mutation of the Sensor Kinase chvG in Rhizobium leguminosarum Negatively Impacts Cellular Metabolism, Outer Membrane Stability, and Symbiosis. J Bacteriol 194: 768–777. 22155778 10.1128/JB.06357-11 PMC3272964

[64] Bladergroen MR, Badelt K, Spaink HP (2003) Infection-blocking genes of a symbiotic Rhizobium leguminosarum strain that are involved in temperature-dependent protein secretion. Mol Plant Microbe Interact 16: 53–64. 12580282 10.1094/MPMI.2003.16.1.53

[65] Viadas C, Rodriguez MC, Sangari FJ, Gorvel JP, Garcia-Lobo JM, et al (2010) Transcriptome analysis of the Brucella abortus BvrR/BvrS two-component regulatory system. PLoS One 5: e10216. 20422049 10.1371/journal.pone.0010216 PMC2858072

[66] Keating DH (2007) The Sinorhizobium meliloti ExoR protein is required for the downregulation of lpsS transcription and succinoglycan biosynthesis in response to divalent cations. FEMS Microbiol Lett 267: 23–29. 17233674 10.1111/j.1574-6968.2006.00498.x

[67] Doherty D, Leigh JA, Glazebrook J, Walker GC (1988) Rhizobium meliloti mutants that overproduce the R. meliloti acidic calcofluor-binding exopolysaccharide. J Bacteriol 170: 4249–4256. 2842307 10.1128/jb.170.9.4249-4256.1988 PMC211434

[68] Martinez-Nunez C, Altamirano-Silva P, Alvarado-Guillen F, Moreno E, Guzman-Verri C, et al (2010) The two-component system BvrR/BvrS regulates the expression of the type IV secretion system VirB in Brucella abortus. J Bacteriol 192: 5603–5608. 20833814 10.1128/JB.00567-10 PMC2953682

[69] Jin LH, Um HJ, Yin CJ, Kim YH, Lee JH (2008) Proteomic analysis of curdlan-producing Agrobacterium sp. in response to pH downshift. J Biotechnol 138: 80–87. 18824044 10.1016/j.jbiotec.2008.08.010

[70] Ingmer H, Brondsted L (2009) Proteases in bacterial pathogenesis. Res Microbiol 160: 704–710. 19778606 10.1016/j.resmic.2009.08.017

[71] Talmadge K, Gilbert W (1982) Cellular location affects protein stability in Escherichia coli. Proc Natl Acad Sci U S A 79: 1830–1833. 7043465 10.1073/pnas.79.6.1830 PMC346074

[72] Shi T, Su D, Liu T, Tang K, Camp DG 2nd, et al (2012) Advancing the sensitivity of selected reaction monitoring-based targeted quantitative proteomics. Proteomics 12: 1074–1092. 22577010 10.1002/pmic.201100436 PMC3375056

[73] Kado CI, Heskett MG (1970) Selective media for isolation of *Agrobacterium, Carynebacterium, Erwinia, Pseudomonas*, and *Xanthomonas* . Phytopathology 60: 969–976. 5469886 10.1094/phyto-60-969

[74] Lai EM, Chesnokova O, Banta LM, Kado CI (2000) Genetic and environmental factors affecting T-Pilin export and T-Pilus biogenesis in relation to flagellation of Agrobacterium tumefaciens. J Bacteriol 182: 3705–3716. 10850985 10.1128/jb.182.13.3705-3716.2000 PMC94541

[75] Quandt J, Hynes MF (1993) Versatile suicide vectors which allow direct selection for gene replacement in gram-negative bacteria. Gene 127: 15–21. 8486283 10.1016/0378-1119(93)90611-6

[76] Vergunst A, Schrammeijer B, den Dulk-Ras A, de Vlaam C, Regensburg-Tuink T, et al (2000) VirB/D4-dependent protein translocation from Agrobacterium into plant cells. SCIENCE 290: 979–982. 11062129 10.1126/science.290.5493.979

[77] Schmidt-Eisenlohr H, Domke N, Baron C (1999) TraC of IncN plasmid pKM101 associates with membranes and extracellular high-molecular-weight structures in Escherichia coli. J Bacteriol 181: 5563–5571. 10482495 10.1128/jb.181.18.5563-5571.1999 PMC94074

[78] Studier FW, Rosenberg AH, Dunn JJ, Dubendorff JW (1990) Use of T7 RNA polymerase to direct expression of cloned genes. Methods Enzymol 185: 60–89. 2199796 10.1016/0076-6879(90)85008-c

[79] Emory SA, Belasco JG (1990) The ompA 5′ untranslated RNA segment functions in Escherichia coli as a growth-rate-regulated mRNA stabilizer whose activity is unrelated to translational efficiency. J Bacteriol 172: 4472–4481. 1695894 10.1128/jb.172.8.4472-4481.1990 PMC213277

[80] Livak KJ, Schmittgen TD (2001) Analysis of relative gene expression data using real-time quantitative PCR and the 2(-Delta Delta C(T)) Method. Methods 25: 402–408. 11846609 10.1006/meth.2001.1262

[81] Lai EM, Kado CI (1998) Processed VirB2 is the major subunit of the promiscuous pilus of Agrobacterium tumefaciens. J Bacteriol 180: 2711–2717. 9573157 10.1128/jb.180.10.2711-2717.1998 PMC107224

[82] Liu AC, Shih HW, Hsu T, Lai EM (2008) A citrate-inducible gene, encoding a putative tricarboxylate transporter, is downregulated by the organic solvent DMSO in Agrobacterium tumefaciens. J Appl Microbiol 105: 1372–1383. 18713283 10.1111/j.1365-2672.2008.03874.x

[83] Baron C, Domke N, Beinhofer M, Hapfelmeier S (2001) Elevated temperature differentially affects virulence, VirB protein accumulation, and T-pilus formation in different Agrobacterium tumefaciens and Agrobacterium vitis strains. J Bacteriol 183: 6852–6861. 11698374 10.1128/JB.183.23.6852-6861.2001 PMC95526

[84] Ho SN, Hunt HD, Horton RM, Pullen JK, Pease LR (1989) Site-directed mutagenesis by overlap extension using the polymerase chain reaction. Gene 77: 51–59. 2744487 10.1016/0378-1119(89)90358-2

