# Supplementary figures and images for: Acid-Induced Type VI Secretion System Is Regulated by ExoR-ChvG/ChvI Signaling Cascade in Agrobacterium tumefaciens
Source: PLoS Pathog. 2012 Sep 27;8(9):e1002938. doi: 10.1371/journal.ppat.1002938 (PMC3460628; doi:10.1371/journal.ppat.1002938)

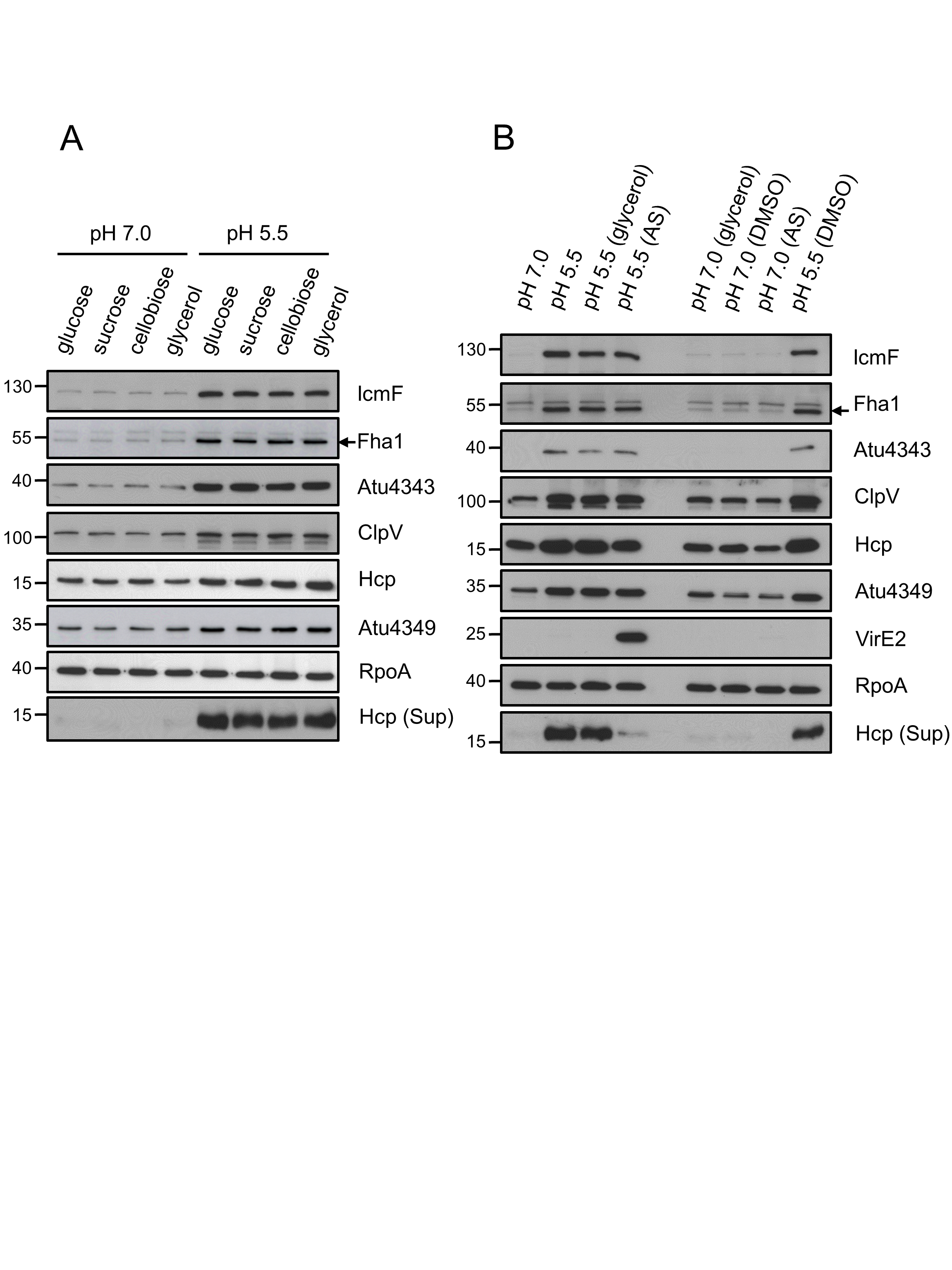

Supplement: Figure S1 — The effects of acetosyringone (AS) and different carbon sources on T6SS expression and Hcp secretion. (A) Total proteins isolated from Agrobacterium tumefaciens wild-type C58 grown in AB-MES (pH 7.0 or pH 5.5) supplemented with indicated carbon sources at 25°C for 6 h were resolved by glycine-SDS-PAGE, followed by western blot analysis with antibodies against C-IcmF, Fha1 (filled arrow), Atu4343, ClpV, Hcp, Atu4349, and RpoA. (B) Total and secreted (Sup) proteins isolated from C58 grown in AB-MES (pH 7.0 or pH 5.5) with glycerol, DMSO (used to dissolve acetosyringone [AS]), or 200 µM AS at 25°C for 6 h were resolved by glycine-SDS-PAGE, followed by western blot analysis with antibodies against C-IcmF, Fha1 (filled arrow), Atu4343, ClpV, Hcp, Atu4349, VirE2, and RpoA. RpoA was used as an internal control. The positions of molecular mass markers (in kDa) are indicated on the left. (TIF) [file ppat.1002938.s001.tif]

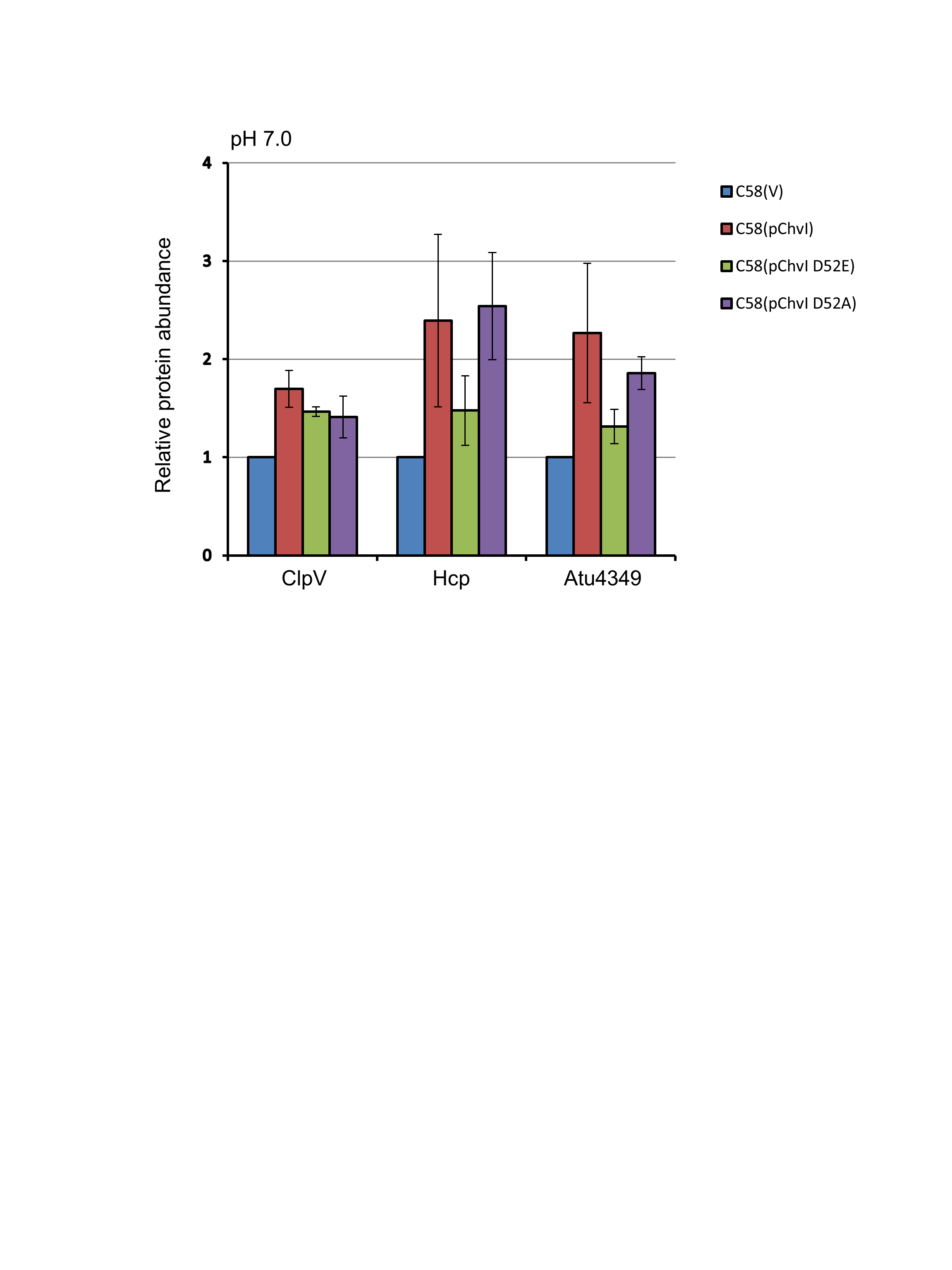

Supplement: Figure S4 — Quantification of levels of hcp operon-encoded proteins with overexpression of ChvI variants. Total proteins isolated from A. tumefaciens C58 strains containing the empty vector (V) or one of the plasmids expressing ChvI, ChvI(D52E) or ChvI(D52A) grown in AB-MES (pH 7.0) at 25°C for 6 h underwent western blot analysis with antibodies against ClpV, Hcp, and Atu4349. Protein levels of ClpV, Hcp, and Atu4349 were quantified with use of the UVP BioSpectrum 600 Imaging System and normalized to the level of endogenous RpoA. The level of vector control was set to 1. Data are mean ± SD of two biological replicates. (TIF) [file ppat.1002938.s004.tif]

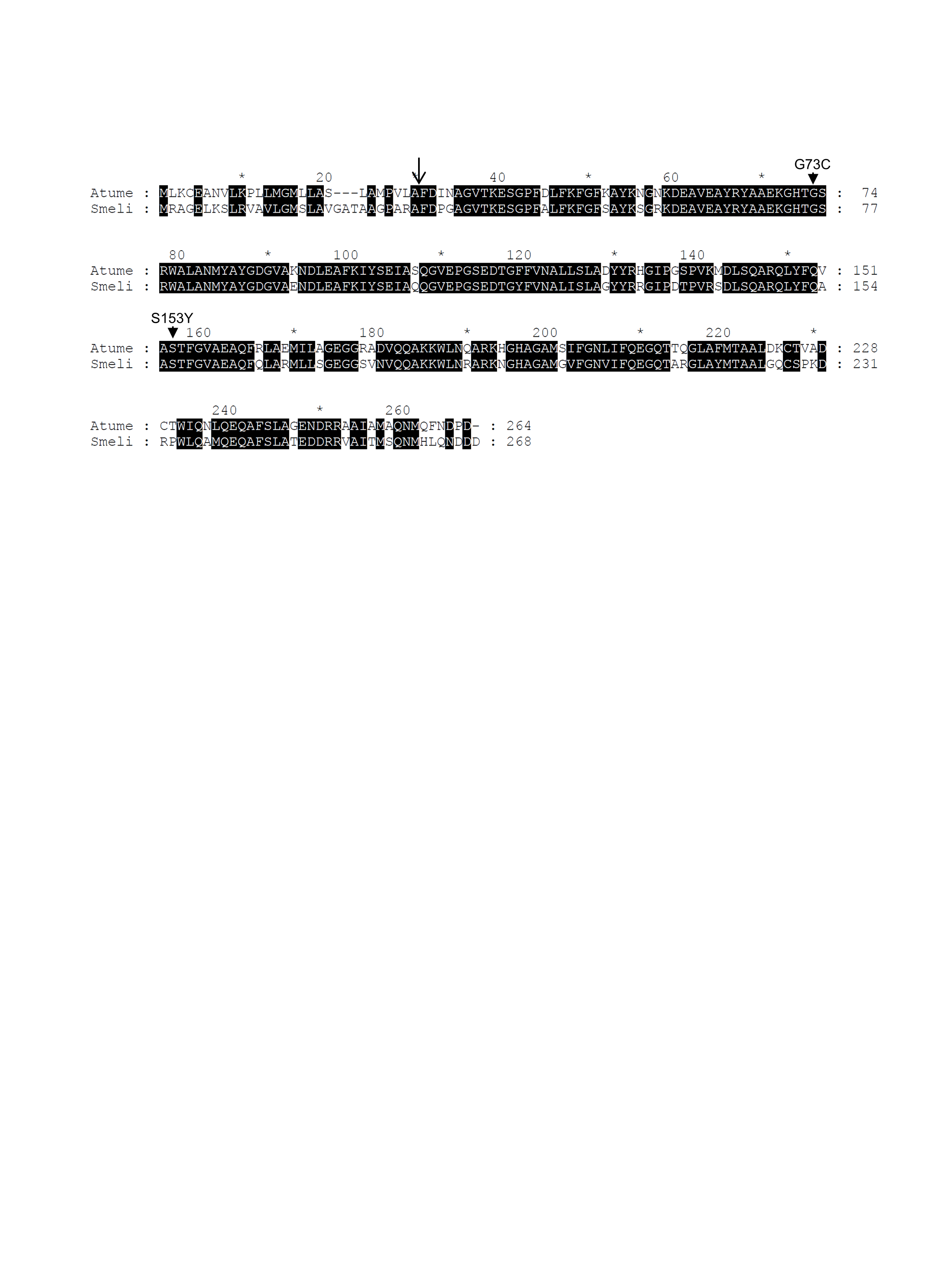

Supplement: Figure S5 — Alignment of the amino acid sequences of A. tumefaciens and S. meliloti ExoR. ExoR orthologs from Atume (A. tumefaciens; Genbank accession no. NP_354703) and Smeli (S. meliloti; AAA26260) were aligned with 74% identity. The identical amino acid residues are highlighted in black, and the arrow indicates a predicted signal peptide cleavage site. The amino acid residues used for mutagenesis are indicated. (TIF) [file ppat.1002938.s005.tif]

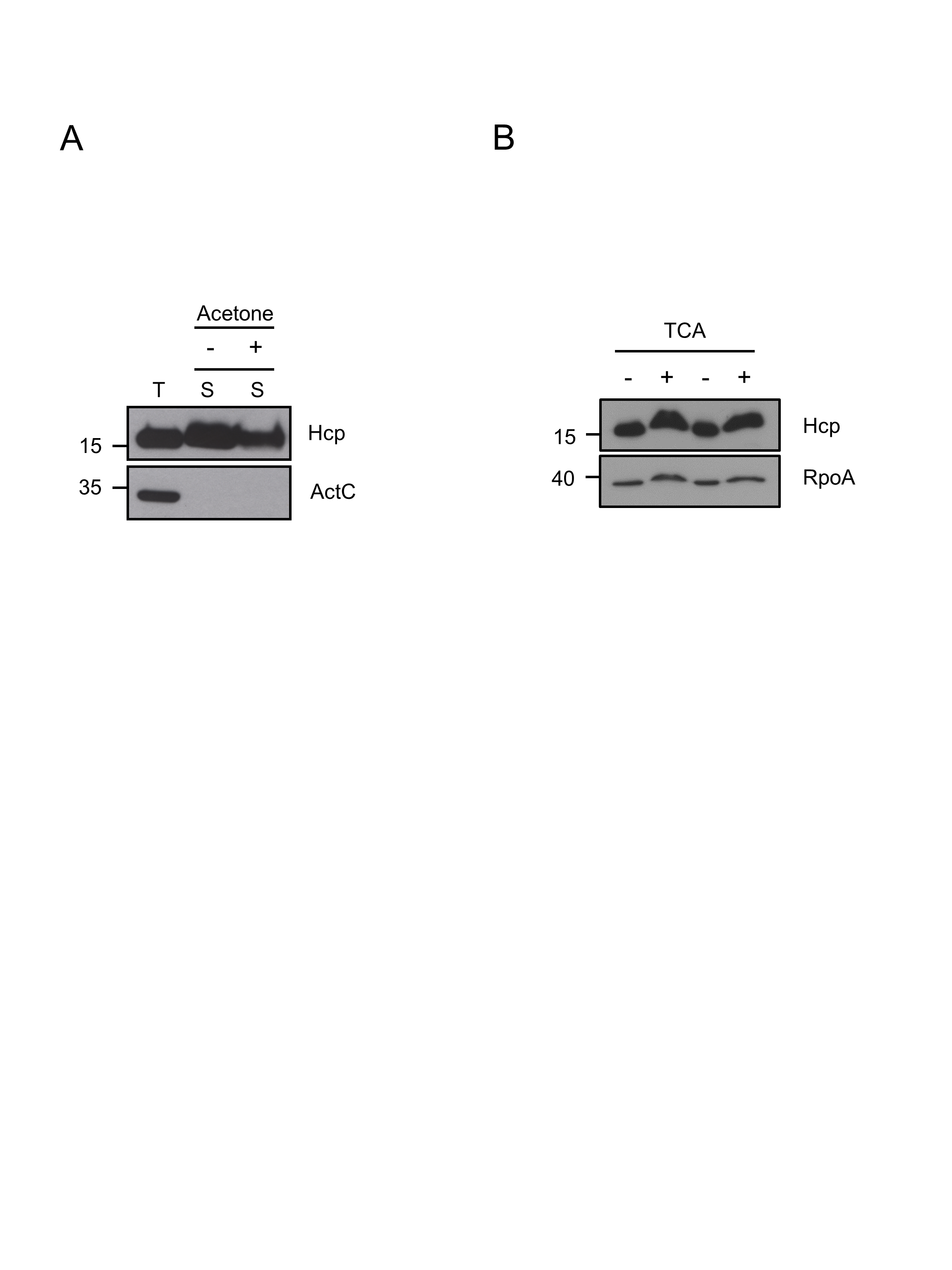

Supplement: Figure S6 — Effect of trichloroacetic acid on migration of cellular and secreted Hcp proteins. (A) Total (T) and secreted (S) proteins were isolated from A. tumefaciens wild-type C58 grown in AB-MES (pH 5.5) at 25°C for 6 h. The supernatant was precipitated by TCA without washing or followed by 85% acetone wash. The proteins were analyzed by western blot analysis with antibodies against Hcp and ActC. The periplasmic protein ActC was an internal control. (B) Western blot analysis of total protein levels of Hcp or RpoA with or without 10% TCA. (TIF) [file ppat.1002938.s006.tif]
