# Supplementary material for: Acid-Induced Type VI Secretion System Is Regulated by ExoR-ChvG/ChvI Signaling Cascade in Agrobacterium tumefaciens
Source: PLoS Pathog. 2012 Sep 27;8(9):e1002938. doi: 10.1371/journal.ppat.1002938 (PMC3460628; doi:10.1371/journal.ppat.1002938)
Supplement: Table S1 — Bacterial strains and plasmids. (PDF) [file ppat.1002938.s008.pdf]

TABLE S1. Bacterial strains and plasmids

Strain or plasmid	Relevant characteristics	Source or Reference
A. tumefaciens strains		
C58	Wild-type virulent strain containing nopaline-type Ti plasmid pTiC58	Eugene Nester
Ach5	Wild-type virulent strain containing octopine-type Ti plasmid pTiAch5	[1]
1D1609	Wild-type virulent strain containing octopine-type Ti plasmid pTi1D1609	[2]
EML1226	*chvG* in-frame deletion mutant, C58Δ*chvG*	This study
EML3682	*chvI* in-frame deletion mutant, C58Δ*chvI*	This study
EML1654	*exoR* in-frame deletion mutant, C58Δ*exoR*	This study
EML2104	*exoR chvG* in-frame deletion mutant, C58Δ*chvG* Δ*exoR*	This study
E. coli strains		
DH10B	Host for DNA cloning	Invitrogen
BL21(DE3)	Host for overexpressing proteins driven by T7 promoter	[3]
S. cerevisiae AH109	Host for yeast two-hybrid analysis	Clontech
Plasmids		
pRL662	Gm^{r}, broad-host-range vector derived from pBBR1MCS-2	[4]
pTrc200	Sm^{r} Sp^{r}, pSV1 origin *lacI*^{q}, *trc* promoter expression vector	[5]
pET22b(+)	Ap^{R}, overexpression vector to generate C-terminal His-tagged protein driven by T7 promoter	Novagen
pET28a	Km^{r}, overexpression vector to generate N-terminal His-tagged protein driven by T7 promoter	Novagen
pJQ200SK	Gm^{r}, suicide plasmid containing Gm^{r} and *sacB* gene for selection of double crossover	[6]
pBluescript SK(+)-HA	pBluescriptsk+::HA for HA epitope tagging vector	This study
pGADT7	Ap^{r}, AD vector used in yeast two-hybrid assay	Clontech
pGBKT7	Km^{r}, DNA-BD vector used in yeast-two hybrid assay	Clontech
pJQ200SK-Δ*chvG*	Gm^{r}, used in generating entire *chvG* deletion mutant of *A. tumefaciens* C58	This study
pJQ200SK-Δ*chvI*	Gm^{r}, used in generating entire *chvI* deletion mutant of *A. tumefaciens* C58	This study
pJQ200SK-Δ*exoR*	Gm^{r}, used in generating entire *exoR* deletion mutant of *A. tumefaciens* C58	This study
pJQ200SK-Δ*exoR-strep*	Gm^{r}, used in generating entire *exoR* replacement with *exoR-strep* of *A.*	This study

	tumefaciens C58	
pET28a-ChvI	Km^r, pET28a expressing His-ChvI	This study
pET28a-ChvI D52E	Km^r, pET28a expressing His-ChvI with D52E substitution	This study
pChvG	Gm^r, pRL662 expressing ChvG driven by *lacZp*	This study
pChvI	Gm^r, pRL662 expressing ChvI driven by *lacZp*	This study
pChvI D52E	Gm^r, pRL662 expressing ChvI with D52E substitution	This study
pChvI D52A	Gm^r, pRL662 expressing ChvI with D52A substitution	This study
pChvG-HA	Gm^r, pRL662 expressing ChvG-HA driven by *lacZp*	This study
pChvI-ChvG-HA	Gm^r, pRL662 expressing ChvI-ChvG-HA driven by native protmoter	This study
pExoR	Sm^r Sp^r, pTrc200 expressing ExoR driven by *trc* promoter	This study
pExoR G73C	Sm^r Sp^r, pTrc200 expressing ExoR with G73C substitution	This study
pExoR S153Y	Sm^r Sp^r, pTrc200 expressing ExoR with S153Y substitution	This study
pExoR G73C S153Y	Sm^r Sp^r, pTrc200 expressing ExoR with G73C and S153Y substitutions	This study
pGBKT7-ChvG-peri	Km^r, DNA-BD vector expressing ChvG periplasmic domain	This study
pGBKT7-53	Km^r, DNA-BD vector expressing murine p53	Clontech
pGADT7-ExoR	Ap^r, AD vector expressing ExoR	This study
pGADT7-ExoR G73C	Ap^r, AD vector expressing ExoR G73C	This study
pGADT7-ExoR S153Y	Ap^r, AD vector expressing ExoR S153Y	This study
pGADT7-ExoR G73C S153Y	AP^r, AD vector expressing ExoR G73C S153Y	This study
pGADT7-T	Ap^r, AD vector expressing SV40 large T-antigen	Clontech

References

1. Lin BC, Kado CI (1977) Studies on *Agrobacterium tumefaciens*. VIII. Avirulence induced by temperature and ethidium bromide. Canadian Journal of Microbiology 23: 1554-1561.

2. Palumbo JD, Phillips DA, Kado CI (1998) Characterization of a new Agrobacterium tumefaciens strain from alfalfa. Arch Microbiol 169: 381-386.

3. Studier FW, Rosenberg AH, Dunn JJ, Dubendorff JW (1990) Use of T7 RNA polymerase to direct expression of cloned genes. Methods Enzymol 185: 60-89.

4. Vergunst A, Schrammeijer B, den Dulk-Ras A, de Vlaam C, Regensburg-Tuink T, et al. (2000) VirB/D4-dependent protein translocation from Agrobacterium into plant cells. SCIENCE 290: 979-982.

5. Schmidt-Eisenlohr H, Domke N, Baron C (1999) TraC of IncN plasmid pKM101 associates with membranes and extracellular high-molecular-weight structures in Escherichia coli. Journal of Bacteriology 181: 5563-5571.

6. Quandt J, Hynes MF (1993) Versatile suicide vectors which allow direct selection for gene replacement in gram-negative bacteria. Gene 127: 15-21.
