# Supplementary material for: Acid-Induced Type VI Secretion System Is Regulated by ExoR-ChvG/ChvI Signaling Cascade in Agrobacterium tumefaciens
Source: PLoS Pathog. 2012 Sep 27;8(9):e1002938. doi: 10.1371/journal.ppat.1002938 (PMC3460628; doi:10.1371/journal.ppat.1002938)
Supplement: Table S2 — Primers used in this study. (PDF) [file ppat.1002938.s009.pdf]

TABLE S2. Primers used in this study

Primer	Sequence (5'-3')	Application
ChvG up F	GCTCTAGAGAAGGATGAGGAGATCGACG	*chvG* deletion
ChvG up R	CGGGATCCCAACACGTATTAAAGTCCTCAC	*chvG* deletion
ChvG down F	CGGGATCCCGCTGAACGCTTCAACCTGC	*chvG* deletion
ChvG down R	TCCCCCCGGGCCTGTCCATCGGTGGGATTTC	*chvG* deletion
ChvI up F	GCTCTAGAGATGGTGTTTTCGGTGGAAGC	*chvI* deletion
ChvI up R	CGGGATCCCTGCATGTAGTTGGTCTCCATC	*chvI* deletion
ChvI down F	CGGGATCCGCTTAAAGAAGCCGACTCAAG	*chvI* deletion
ChvI down R	TCCCCCCGGGAACAGATTGGCATCGGCATCG	*chvI* deletion
ExoR up F	GCTCTAGAATGCGCGACTATGATTCCTGC	*exoR* deletion
ExoR up R	CGGGATCCCAGCATTTCGGCGAACTTCTC	*exoR* deletion
ExoR down F	CGGGATCCGATTGAGAATGCCGGTCGGGAC	*exoR* deletion
ExoR down R	TCCCCCCGGGGCCTTCCGCAAACTGGAAAATC	*exoR* deletion
ExoR-strep down F	TGAGAATGCCGGTCGGGACGGTTTCA	*exoR-strep* replacement
ExoR-strep down R	TGAAACCGTCCCGACCGGCATTCTCACTTTTCGAACTGCGGGTGGCTCCA ATCCGGATCGTTGAACTGCATG	*exoR-strep* replacement
ExoR-strep F	CTCTCGCCGGTGAAAACGACC	confirmation
ExoR-strep R	GCTCTAGATCGGCTGAACCGTTGCTGTC	confirmation
T6SS F	GGGGTACCGACATGCAATGAGCCTCCTGC	EMSA
T6SS R	CCGCTCGAGGAGTAGTCTATCCCCAGAAAATAG	EMSA
Atu4353 F	TCCGCATCCATGGGAATAGC	EMSA
Atu4353 R	CGGGATCCTTCCGTCGCGTATATCCACG	EMSA
PET28a-ChvI F	AAACATATGCAGACGATCGCGCTTGTC	Purification
PET28a-ChvI R	CCGCTCGAGTTAAGCCGCTTCGCGGAAG	Purification
ChvG F	CCGCTCGAGGAAGCCGACTCAAGCAGAAG	overexpression
ChvG R	GCTCTAGAGGTCGCATGCAGGTTGAAGC	overexpression
ChvI F	CCGCTCGAGAACCTGGCTGGGTGAATGTCG	overexpression
ChvI R	GCTCTAGAGGTCCCTTCTGCTTGAGTCG	overexpression
ChvG-HA F	GGAGCTCCCGCTCGAGGAAGCCGACTCAAGCAGAAG	overexpression
ChvG-HA R	GCTCTAGAGCGTTCATGGGTTTCGGCCG	overexpression
ChvI-ChvG-HA F	GCTCTAGAGAGCCTGACTTTAAAAAACTTAG	overexpression
ChvI-ChvG-HA R	CCGCTCGAGTCAAGCGTAATCTGGAACATCGTATGGGTAGCGTTCATGG GTTTCGGCCG	overexpression
ExoR F	GCCCGGGAGTGAGAGAAGTTCGCCGAAATGC	overexpression
ExoR R	GATTCTAGATCAATCCGGATCGTTGAACTGC	overexpression
ChvI D52E F	GCTTGCCATTTTC**GAA**ATTAAGATGC	mutagenesis

ChvI D52E R	GCATCTTAAT**TTC**GAAAATGGCAAGC	mutagenesis
ChvI D52A F	GCTTGCCATTTTC**GCT**ATTAAGATGC	mutagenesis
ChvI D52A R	GCATCTTAAT**AGC**GAAAATGGCAAGC	mutagenesis
ExoR G73C F	AGGGCCATACC**TGC**TCGCGCTGG	mutagenesis
ExoR G73C R	CCAGCGCGA**GCA**GGTATGGCCCT	mutagenesis
ExoR S153Y F	CCAGGTGGCT**TAC**ACCTTTGGCGTG	mutagenesis
ExoR S153Y R	CACGCCAAAGGT**GTA**AGCCACCTGG	mutagenesis
icmF F	CGGTTGTTCAAGGACGTCATC	qRT-PCR
icmF R	GCGGATACGGCATAAGCAAT	qRT-PCR
fha1 F	GCGACCACGAACATACCGAT	qRT-PCR
fha1 R	GACCGGACGTGAGATCATGAC	qRT-PCR
atu4343 F	TTCTTTCGGTTGCGCGTTAC	qRT-PCR
atu4343 R	GAACCAGCGTTTCGATGGAG	qRT-PCR
clpV F	AGACGTTGCCAGAGCGGTTA	qRT-PCR
clpV R	ACAGAAGGTGTCGCTCGCTT	qRT-PCR
hcp F	CGTTCGACGACAACAACTCG	qRT-PCR
hcp R	GTCGCCAGATCGTAGGAAGC	qRT-PCR
atu4349 F	GAAAGCTGCGATGTTGCATTC	qRT-PCR
atu4349 R	GGTGGAGTATTCGCTTTCAGGA	qRT-PCR
16S F	ACCCATCTCTGCGGAATAGCT	qRT-PCR
16S R	GCTCATCCATCCCCGATAAAT	qRT-PCR
23S F	CGTCTCTGCTCGACTTGTCAGT	qRT-PCR
23S R	AATCGGTCGTCGAGTGCAAT	qRT-PCR
chvH F	TCCATTCACGAAGGCAATCC	qRT-PCR
chvH R	ACGATTTCCAGCGTGACATG	qRT-PCR
BD ChvGperi F	GAACATATGGAAGGGCTGATCGACGCGCGCGTG	Yeast 2 hybrid
BD ChvGperi R	CCGCTCGAGCGCGCTCGGCATGCACGATCTTG	Yeast 2 hybrid
AD ExoR F	GACGGATCCATCCGGATCGTTGAACTGCATG	Yeast 2 hybrid
AD ExoR R	GACGAATTCATCCGGATCGTTGAACTGCATG	Yeast 2 hybrid

[a] Restriction enzyme sites are underlined, and mutated sequences are indicated by bold type.
